# Glutamatergic neurotransmission in schizophrenia: A systematic review and quantitative synthesis of proton magnetic resonance spectroscopy studies across schizophrenia spectrum disorders

**DOI:** 10.1177/00048674241254216

**Published:** 2024-05-29

**Authors:** Jamie J Lopes, Sean P Carruthers, Denny Meyer, Brian Dean, Susan L Rossell

**Affiliations:** 1 Centre for Mental Health, Swinburne University of Technology, Melbourne, VIC, Australia; 2 Molecular Psychiatry Laboratory, The Florey Institute of Neuroscience and Mental Health, Melbourne, VIC, Australia; 3 Department of Psychiatry, St Vincent’s Hospital, Melbourne, VIC, Australia

**Keywords:** Glutamate, glutamine, schizophrenia, magnetic resonance spectroscopy

## Abstract

**Objective:**

Studies using proton magnetic resonance spectroscopy reveal substantial inconsistencies in the levels of brain glutamate, glutamine and glutamate + glutamine across schizophrenia spectrum disorders. This systematic review employs qualitative and quantitative methods to analyse the patterns and relationships between glutamatergic metabolites, schizophrenia spectrum disorders and brain regions.

**Methods:**

A literature search was conducted using various databases with keywords including glutamate, glutamine, schizophrenia, psychosis and proton magnetic resonance spectroscopy. Inclusion criteria were limited to case-control studies that reported glutamatergic metabolite levels in adult patients with a schizophrenia spectrum disorder diagnosis – i.e. first-episode psychosis, schizophrenia, treatment-resistant schizophrenia and/or ultra-treatment-resistant schizophrenia – using proton magnetic resonance spectroscopy at 3 T or above. Pooled study data were synthesized and analysed.

**Results:**

A total of 92 studies met the inclusion criteria, including 2721 healthy controls and 2822 schizophrenia spectrum disorder participants. Glu levels were higher in the basal ganglia, frontal cortex and medial prefrontal of first-episode psychosis participants, contrasting overall lower levels in schizophrenia participants. For Gln, strong differences in metabolite levels were evident in the basal ganglia, dorsolateral prefrontal cortex and frontal cortex, with first-episode psychosis showing significantly higher levels in the basal ganglia. In glutamate + glutamine, higher metabolite levels were found across schizophrenia spectrum disorder groups, particularly in the basal ganglia and dorsolateral prefrontal cortex of treatment-resistant schizophrenia participants. Significant relationships were found between metabolite levels and medication status, clinical measures and methodological variables.

**Conclusion:**

The review highlights abnormal glutamatergic metabolite levels throughout schizophrenia spectrum disorders and in specific brain regions. The review underscores the importance of standardized future research assessing glutamatergic metabolites using proton magnetic resonance spectroscopy due to considerable literature heterogeneity.

## Introduction

The glutamate hypothesis for schizophrenia asserts that glutamatergic dysfunction is a key causal contributor to both the pathogenesis and the symptomatology of schizophrenia (Egerton and Stone, 2012). Advances in neuroimaging techniques have permitted comprehensive testing of this hypothesis via examination of the extent of glutamatergic dysfunction in people living with schizophrenia spectrum disorders (SSD) and the corresponding clinical consequences. Despite the prominence of the glutamate hypothesis, ongoing research on the role of brain glutamate in schizophrenia using proton magnetic resonance spectroscopy (^1^H-MRS), although promising, has not produced robust evidence to support the hypothesis. Several systematic reviews and meta-analyses have been conducted on ^1^H-MRS studies assessing glutamatergic metabolites across the clinical spectrum of schizophrenia (Iwata et al., 2018; Kumar et al., 2020; Marsman et al., 2013; Merritt et al., 2016, 2021; Nakahara et al., 2022; Poels et al., 2014; Salavati et al., 2015; Smucny et al., 2021). The brain areas most consistently affected appear to be the anterior cingulate cortex (ACC), the basal ganglia, the midcingulate cortex and the medial prefrontal (MPF) cortex. Glutamatergic metabolite levels, however, do not present a uniform pattern across brain areas or SSD. A summary of these findings can be found in Table 1.

**Table 1.** Summary of ^1^H-MRS glutamatergic findings.

SSD	Glu	Gln	Glx
FEP	↑ BG, MPF ↓ MCC	–	↑ BG, DLPFC, Hipp
Schizophrenia	↑ BG ↓ MCC, MFC, MPF	↑ ACC, MFC, PFC, Striatum, Thalamus	↑ BG, OCC, PC, TC ↓ MFC
TRS + uTRS	↑ ACC, MCC	–	↑ ACC, MCC

↑: Higher levels; ↓: lower levels; ACC: anterior cingulate cortex; BG: basal ganglia; DLPFC: dorsolateral prefrontal cortex; FEP: first-episode psychosis; Gln: glutamine; Glu: glutamate; Glx: Glu + Gln; Hipp: hippocampus; MCC: midcingulate cortex; MFC: medial frontal cortex; MPF: medial prefrontal region; OCC: occipital cortex; PFC: prefrontal cortex; ^1^H-MRS: proton magnetic resonance spectroscopy; SSD: schizophrenia spectrum disorders; TRS: treatment-resistant schizophrenia; uTRS: ultra-TRS.

The table provides a summary of the outcomes obtained from meta-analyses and systematic reviews investigating glutamatergic metabolites using ^1^H-MRS studies across SSD (Iwata et al., 2018; Kumar et al., 2020; Marsman et al., 2013; Merritt et al., 2016, 2021; Nakahara et al., 2022; Poels et al., 2014; Salavati et al., 2015; Smucny et al., 2021).

Contributing to this complex pattern of results is the considerable methodological diversity in the field, particularly regarding MRI field strength. Both glutamate (Glu) and glutamine (Gln) have similar chemical compositions and narrow chemical shift ranges which often leads to significant MRS spectral overlap between the two metabolite resonances (i.e. Glx), with greater contamination taking place at lower field strengths (Snyder and Wilman, 2010). Higher field strengths (e.g. 3 T and above) increase proton polarization and, thus, signal intensity, which allows for a higher degree of spectral resolution and provides better separation of metabolite signals from background noise (Snyder and Wilman, 2010). For this reason, the current systematic review will collate all ^1^H-MRS findings to date using a field strength of 3 T or above used to investigate glutamatergic metabolite (i.e. Glu, Gln and Glx) levels across four distinct SSD groups (i.e. first-episode psychosis [FEP], schizophrenia, treatment-resistant schizophrenia [TRS] and ultra-TRS [uTRS]) across varying brain regions.

Though valuable, meta-analyses can often fail to capture nuances present in a vast and heterogeneous range of studies. Previous meta-analyses have highlighted the significant discrepancy between studies, particularly the small sample sizes, varying brain regions, variability among patient diagnoses, differing ^1^H-MRS methodologies, difficulty discerning between Glu and Gln at lower field strengths, etc. (Iwata et al., 2018; Kumar et al., 2020; Marsman et al., 2013; Merritt et al., 2016, 2021; Nakahara et al., 2022; Poels et al., 2014; Salavati et al., 2015; Smucny et al., 2021). Employing a meta-analytical method that solely combines data from various studies might not be optimal, potentially missing crucial findings, overlooking nuanced variations and diminishing their impact. Given the discrepancy in findings and the vast dataset, a standard systematic qualitative synthesis inadequately assesses glutamatergic neurometabolite levels in schizophrenia. This systematic review employs a mixed-methods approach, combining quantitative and qualitative analyses. Pooled effect sizes (Cohen’s *d*) will be calculated to estimate the overall effect of glutamatergic metabolite levels between SSD groups and healthy controls (HC) across brain regions. In addition, a qualitative synthesis will explore patterns and trends in findings to provide context to the quantitative results. The impact of medication status and methodological variables on metabolite quantification will also be assessed. This approach addresses limitations in previous work by providing a multidirectional assessment of the evidence related to the role of glutamatergic neurometabolites in schizophrenia. While prior reviews included research up to November 2020 (Nakahara et al., 2022), this review distinguishes itself by incorporating the latest research up to June 2022 with studies not included in previous reviews, thereby expanding the timeframe and collective sample size with an additional 447 SSD participants and 414 HC.

## Methods

### Data sources and search strategy

This systematic review was conducted in accordance with Preferred Reporting Items for Systematic reviews and Meta-Analyses (PRISMA) guidelines. The systematic review protocol for this work was published as a preprint in PsyArXiv Preprints in August 2020 (Lopes et al., 2020). Deviations from the review protocol were: (1) to allow comparisons between participants within an SSD and HC, only studies with data on these two groups were included, (2) for longitudinal studies, only the first timepoint data was included, (3) where the same participant data was used in different studies only the publication with the largest reported sample size was included and (4) in studies with mixed diagnoses only data from the diagnostic groups included in our analysis were included.

A systematic computerized literature search of articles up to 28 June 2022 was conducted using Web of Science, PsychNet, EBSCOhost and PubMed. Search terms were selected to represent schizophrenia throughout its phenotypic presentations, Glu and Gln and ^1^H-MRS. These search terms were: (glutamate OR glutamine) AND (schizo* OR psychosis) AND (MRS OR ‘magnetic resonance spectroscopy’ OR ‘MR spectroscopy’) AND (patient OR participant OR people OR person).

### Selection criteria

Studies were included if (1) they were part of an original empirical investigation, containing (2) adult human participant samples, of which (3) one or more participant groups had a diagnosis of FEP, schizophrenia, schizoaffective disorder or schizophreniform disorder, whose (4) brain metabolite concentration data included Glu and/or Gln (5) that was collected using ^1^H-MRS. Exclusion criteria included (1) animal studies, (2) case studies, (3) studies using post-mortem tissue and (4) ^1^H-MRS field strengths below 3 T. The authors were contacted for this information if the above criteria were not made explicit in the study. Studies were excluded if they were not case-controlled to include HC and if they did not report on independent glutamatergic statistical data. Inclusion was limited to studies performed in patients with FEP, schizophrenia, TRS and/or uTRS; studies were excluded if participant diagnostic information was not discriminated against or if collated ^1^H-MRS data pertained to mixed diagnoses.

### Data collection

**Figure 1.**
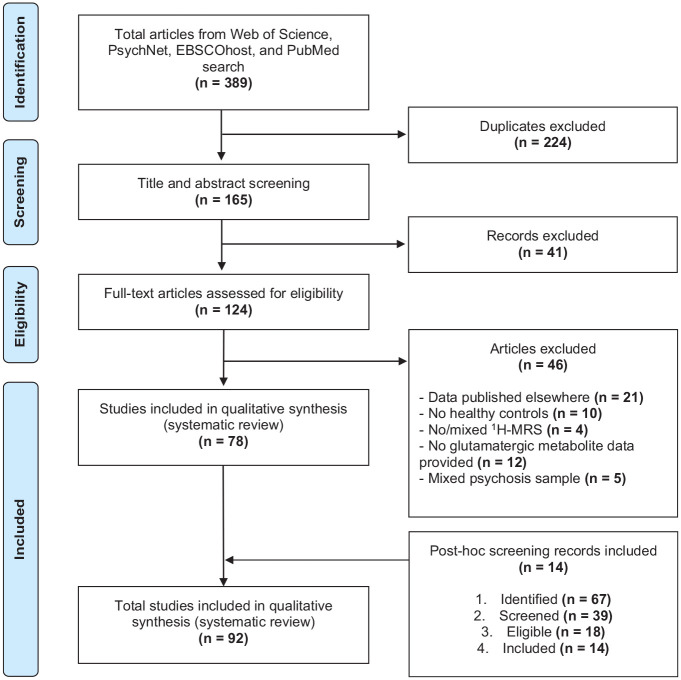
PRISMA flow diagram for literature search and exclusion process.

The selection procedure is illustrated in Figure 1 and followed PRISMA standards (Moher et al., 2009). A two-staged screening process was employed to determine record eligibility and was outlined in a preprint (Lopes et al., 2020). Duplicate articles were removed. Possible articles were initially screened independently (J.J.L.) for inclusion through reading article title, abstract and keyword screening. The full-text screening was then conducted independently by two assessors (J.J.L. and S.P.C.) for studies potentially meeting the inclusion criteria; discrepancies between reviewers were either resolved by consensus or arbitrated by a third reviewer (S.L.R.), where necessary.

### Data extraction

Statistical data for clinical participants and HC, in terms of their glutamatergic metabolite concentrations, were extracted from the main text or Supplemental Materials. Statistical data included sample size (*n*), means ( $\bar{x}$ ) and standard deviations (*s*) for ^1^H-MRS Glu, Gln and/or Glx concentrations; only ^1^H-MRS participants were reported in sample size. From this two-sample data, calculations were made for degrees of freedom (*df*; *df* = *n*_1_+*n*_2_ – 2), t-values (*t*; $t=({\bar{x}}_{1}-{\bar{x}}_{2})/\sqrt{(s_{1}^{2}}/n_{1}+s_{2}^{2}/n_{2})$ , Cohen’s *d* effect sizes (*d*; $d=t\sqrt{(1/n_{1}}+1/n_{2})$ ) and probability values (*p*) using the T.DIST.2T function in Microsoft Excel. Where means and standard deviations were not made available, reported t-values were used to calculate probability values and effect sizes. In cases where two-group comparisons reported F-scores (*F*), these were converted to t-values ( $t=\sqrt{F}$ ).

Extracted data also included the SSD group, brain region investigated, specific glutamatergic metabolite(s) studied and ^1^H-MRS acquisition details, including magnetic field strength (T), echo time (TE), voxel size and ^1^H-MRS sequence. Data pertaining to participant clinical measures (i.e. medication status and symptom rating scales) were also included in this review. These included participant antipsychotic status and, where possible, chlorpromazine equivalent dose, the Positive and Negative Symptoms Scale (PANSS), the Scale for the Assessment of Negative Symptoms (SANS), the Scale for the Assessment of Positive Symptoms (SAPS) and the Brief Psychiatric Rating Scale (BPRS).

Statistical significance was noted, and Cohen’s *d* effect sizes were calculated for each between-group comparison (i.e. between SSD participants and HC), representing the magnitude of difference in glutamatergic metabolite levels between SSD participants and HC. For this analysis, a significance level of < 0.05 was used to determine the statistical significance of a study. Effect size was calculated using Cohen’s *d*, where standardized mean differences of 0.2, 0.5 and 0.8 correspond to small, medium and large effect sizes, respectively, while values below 0.2 were deemed negligible.

### Statistical analyses

Cohen’s *d* was used to measure the effect size of glutamatergic metabolite concentration differences between SSD participants and HC. For the quantitative analyses of this review, Cohen’s *d* was chosen as the dependent variable due to it being a standardized measure of the strength of a relationship, independent of sample sizes. Furthermore, to account for the potential increased reliability of studies with larger sample sizes, the analyses in this review employed SSD group participant sample sizes as a weighting factor.

Kolmogorov–Smirnov tests revealed the Cohen’s *d* values for the metabolite concentrations across brain region and SSD groups were not normally distributed (*p* < 0.001). As such, this review employed non-parametric tests for the following analyses. Kruskal–Wallis tests were used to assess the relationship between Cohen’s *d* and SSD groups, brain regions and medication status, with separate analyses for Glu, Gln and Glx; Spearman’s correlation coefficients were used to explore the strength and direction of association between clinical measures and chlorpromazine equivalent doses with effect size. Chi-square tests of independence were used to examine the relationships between echo time, field strength and ¹H-MRS sequence and effect size (Cohen’s *d*). Echo times were categorized into short (TE < 30 ms), medium (30 ms ⩽ TE < 80 ms) and long (TE ⩾ 80 ms), while effect sizes were categorized into direction and magnitude in the following manner: negative large (*d* ⩽ −0.80), medium (−0.80 < *d* ⩽ −0.50) and small (*d* ⩽ −0.20), null (−0.20 < *d* < 0.20) and positive small (*d* ⩽ 0.20), medium (0.50 ⩽ *d* < 0.80) and large (*d* ⩾ 0.80). Pearson’s chi-square test and Cramer’s V were used to assess the association, with adjusted standardized residuals exceeding ± 3.2 used to identify significant contributions in the contingency table.

Unless otherwise stated, the above analyses were performed using IBM SPSS Statistics (Version 29). Effect size values indicate the direction and magnitude of glutamatergic metabolite level differences in participants within an SSD group with respect to HC.

The findings of this review are presented in three parts: first, a summary of the key characteristics of the reported studies; second, a qualitative analysis of the collected data, highlighting emerging trends and patterns and third, the above quantitative statistical analyses are performed and the relationships with Cohen’s *d* are reported.

## Results

The literature and cross-reference searches initially returned 389 records which matched the search criteria. After the removal of duplicates, screening and assessment for eligibility, a total of 92 studies met our inclusion criteria and were included in the review (Figure 1). From the 92 studies, 11 studies did not provide participant means and standard deviations (Aoyama et al., 2011; Briend et al., 2020; Bustillo et al., 2014; Cadena et al., 2018; Chang et al., 2007; Gurler et al., 2021; Hugdahl et al., 2015; Jauhar et al., 2018; Rauchmann et al., 2020; Shakory et al., 2018; Théberge et al., 2003). While some studies assessed Glu, Gln and Glx in isolation within specific brain regions, the majority assessed multiple metabolites (e.g. creatine, *N*-acetylaspartate, myo-inositol) and voxel placements as part of one study. Many compared SSD and healthy control groups, leading to the number of finding reports being greater than the number of studies.

A total of 330 ^1^H-MRS between-group comparisons were found, of which 265 reported statistically significant differences between SSD and healthy control participants. Notably, nine of the included studies (Bryant et al., 2021; Ćurčić-Blake et al., 2017; Kaminski et al., 2020; Kegeles et al., 2012; Leptourgos et al., 2022; Rowland et al., 2013; Shakory et al., 2018; Tayoshi et al., 2009; Wang et al., 2022) categorized SSD participants based on demographic characteristics, such as gender and age (e.g. male vs female, young vs old, etc.). For the purpose of this review, these subgroup analyses were integrated into their respective broader SSD group (e.g. FEP), resulting in an adjusted total of 304 between-group comparisons. Effect size was calculated for all but one between-group comparison where no relevant statistical data were reported (Théberge et al., 2003).

### Study characteristics

Thirty-one studies recruited participants with FEP, 61 with schizophrenia, 5 with TRS and 2 with uTRS. In these studies, a total of 125 between-group comparisons were obtained for Glu levels, 47 for Gln levels and 132 for Glx levels; from these, 26, 2 and 39 normalized Glu, Gln and Glx, respectively, to creatine. Neuroanatomical regions of interest included: ACC, basal ganglia (including the associative striatum, caudate, putamen, striatum and substantia nigra), centrum semiovale, cerebellum, dorsolateral prefrontal cortex (DLPFC), frontal lobe (including the frontal cortex, frontal white matter [WM], medial frontal cortex [MFC] and orbitofrontal region), inferior frontal gyrus (IFG), insula, MPF (including the prefrontal cortex [PFC], medial PFC, prefrontal WM and ventromedial PFC), the temporal lobe (including the auditory complex, hippocampus, superior temporal gyrus [STG], superior temporal sulcus [STS], temporal cortex and temporal WM), thalamus and visual cortex (including the occipital cortex [OCC], OCC WM, parietal cortex and parieto-occipital cortex [POC]).

Sample sizes of included participants ranged from 6 to 114 for the patient group and 10 to 89 for HC. Seventy-four studies were performed at a magnetic field strength of 3 T, 6 at 4 T and 12 at 7 T. Regarding ^1^H-MRS sequences methods, point-resolved spectroscopy (PRESS) was used in 61 studies, stimulated echo acquisition mode (STEAM) in 17 studies, Mesher-Garwood (MEGA)-PRESS in 10 studies, semi-localized by adiabatic selective refocusing (sLASER) in three studies and spin echo full intensity acquired localized (SPECIAL) pulse sequence and MEGA-sLASER were used in one study each. Echo times varied between 5 and 500 ms. Study characteristics are provided in Table 2.

**Table 2.** Study characteristics.

First author	Year	SSD	T	Sequence	TE	Brain region	Metabolite	n SSD	n HC	Result	Cohen’s *d*
Aoyama	2011	Sz	4	STEAM	20	ACC	Gln	13	17	NS	0.83^a^
						Thalamus		15	17	+	0.96
Atagun	2015	Sz	3	PRESS	30	ST gyrus	Glu	30	30	–	−0.58
Balz	2018	Sz	3	SPECIAL	8.5	ST sulcus	Glu	15	19	+	0.72
						OCC		15	19	NS	0.01
Bartolomeo	2020	FEP	3	PRESS	30	MFC	Glx	34	19	+	0.67
Birur	2020	FEP	3	PRESS	80	MPFC	Glx	20	18	NS	0.00
Bojesen	2021	Sz	3	MEGAPRESS	68	ACC	Glx	37	47	NS	−0.41
				PRESS	30	ACC	Glu	48	51	NR	−0.20
							Gln	40	40	NS	0.07
							Glx	48	51	NR	−0.09
						Thalamus	Glu	48	46	+	0.26
							Glx	48	47	NS	0.07
Borgan	2020	FEP	3	PRESS	30	ACC	Glu	20	20	NS	0.52
Brandt	2016	Sz	7	STEAM	28	ACC	Glu	24	24	NS	0.09
							Gln	24	24	NS	−0.17
							Glx	24	24	NS	−0.46
Briend	2020	FEP	3	PRESS	80	Hippocampus	Glx	54	41	NS	−0.18^a^
Bustillo	2014	Sz	3	PRESS	40	ACC	Glu	84	81	NS	0.02^a^
							Gln	72	76	+	0.41
							Glx	84	81	+	0.43
Bustillo	2010	Sz	4	STEAM	20	ACC	Glu	14	8	NS	−0.57
							Gln	14	8	NS	1.01
							Glx	14	8	+	1.44
						Thalamus	Glu	12	10	NS	0.20
							Gln	12	10	NS	−0.09
							Glx	12	10	NS	0.19
						Frontal WM	Glu	10	10	NS	−0.12
							Gln	10	10	NS	0.66
							Glx	10	10	NS	0.67
Byrant	2021	FEP	3	PRESS	30	Frontal WM	Glu	57	57	NR	0.34
Cadena	2018	Sz	3	PRESS	80	ACC	Glx/Cr	22	20	+	0.64^a^
Cen	2020	FEP	3	MEGAPRESS	68	MPFC	Glx	23	26	NS	0.27
Chang	2007	Sz	4	PRESS	30	Total	Glx	23	22	+	1.00^a^
						Frontal WM		23	22	NR	0.00
						Temporal WM		23	22	NR	0.00
						OCC WM		23	22	NR	0.00
Chiappelli	2018	Sz	3	STEAM	6.5	ACC	Glu	56	58	NS	−0.56
								56	58	–	−0.38
Chiappelli	2015	Sz	3	PRESS	30	Frontal WM	Glu	38	36	NS	−0.05
Chiu	2018	FEP	3	MEGAPRESS	68	ACC	Glx	19	14	+	2.11
Corcoran	2020	Sz	3	PRESS	80	ACC	Glu	20	28	NS	−0.23
						R DLPFC		18	33	NS	−0.19
						L DLPFC		20	34	–	−0.67
Coughlin	2020	Sz	3	PRESS	35	ACC	Glx/Cr	22	13	NS	−0.43
Coughlin	2015	Sz	3	PRESS	35	ACC	Glx/Cr	25	17	NS	−0.19
						DLPFC		25	16	NS	0.10
Crocker	2014	FEP	3	STEAM	240	MPFC	Glu	29	45	NS	0.17
Curcic-Blake	2017	Sz	3	PRESS	144	PFC	Glx	67	30	–	−0.69
de la Fuente-Sandoval	2018	FEP	3	PRESS	68	MPFC	Glx	26	17	+	0.82
						Caudate		27	17	+	0.83
de la Fuente-Sandoval	2013	FEP	3	PRESS	35	Caudate	Glu	24	18	+	0.86
							Glx	24	18	NS	0.68
						Cerebellum	Glu	24	18	+	0.66
							Glx	24	18	NS	0.05
de la Fuente-Sandoval	2011	FEP	3	PRESS	35	Caudate	Glu	18	40	NS	0.93
							Glx	18	40	NS	0.62
						Cerebellum	Glu	18	40	+	0.65
							Glx	18	40	+	0.71
Demjaha	2014	TRS	3	PRESS	30	ACC	Glu	6	10	+	1.33
							Glx	6	10	NS	0.64
		Sz	3	PRESS	30	ACC	Glu	8	10	NS	0.13
							Glx	8	10	NS	0.48
Dempster	2020	FEP	7	sLASER	100	ACC	Glu	26	27	NS	0.07
Egerton	2018	Sz	3	PRESS	30	ACC	Glu/Cr	26	36	NS	0.00
							Glx/Cr	26	36	NS	−0.16
						Thalamus	Glu/Cr	24	36	NS	−0.40
							Glx/Cr	26	36	NS	−0.20
						ACC	Glu/Cr	31	15	NS	0.08
							Glx/Cr	31	15	NS	0.30
						Thalamus	Glu/Cr	27	14	NS	−0.45
							Glx/Cr	27	14	NS	0.09
						ACC	Glu/Cr	13	9	NS	−0.71
							Glx/Cr	13	9	NS	−0.57
						Thalamus	Glu/Cr	11	9	NS	0.77
							Glx/Cr	12	7	NS	0.43
Falkenberg	2014	Sz	3	PRESS	35	L ACC	Glu/Cr	17	17	–	−0.91
						R ACC		17	17	NS	−0.05
Gallinat	2016	Sz	3	PRESS	80	ACC	Glu	29	29	–	−0.52
						Hippocampus		29	27	+	1.12
Girgis	2019	Sz	3	PRESS	139	ACC	Glu	19	20	NS	−0.07
							Glx	19	20	NS	−0.56
						MPFC	Glu	19	20	NS	−0.08
							Glx	19	20	NS	−0.39
Godlewska	2021	FEP	7	STEAM	11	ACC	Glu	14	18	–	−3.02
							Gln	14	18	–	−3.85
						DLPFC	Glu	14	16	NS	−0.28
							Gln	14	16	NS	−2.60
						Putamen	Glu	16	18	NS	−0.42
							Gln	16	18	NS	1.22
Goldstein	2015	Sz	3	PRESS	30	DLPFC	Glu/Cr	15	16	NR	2.20
							Glx/Cr	15	16	NS	3.33
						ACC	Glu/Cr	14	13	NR	−0.35
							Glx/Cr	14	13	NS	−0.54
						Putamen	Glu/Cr	12	13	NR	−0.31
							Glx/Cr	12	13	NS	0.59
		TRS	3	PRESS	30	DLPFC	Glu/Cr	16	16	NR	1.00
							Glx/Cr	16	16	NS	1.17
						ACC	Glu/Cr	14	13	NR	−1.56
							Glx/Cr	14	13	NS	−1.69
						Putamen	Glu/Cr	8	13	NR	0.41
							Glx/Cr	8	13	NS	3.63
		uTRS	3	PRESS	30	DLPFC	Glu/Cr	11	16	NR	−0.71
							Glx/Cr	11	16	NS	−0.91
						ACC	Glu/Cr	9	13	NR	−0.40
							Glx/Cr	9	13	NS	−0.66
						Putamen	Glu/Cr	9	13	NR	−0.52
							Glx/Cr	9	13	NS	−0.52
Goto	2012	FEP	3	MEGAPRESS	68	Frontal lobe	Glx/Cr	18	18	NS	0.35
						Basal ganglia	Glx/Cr	18	18	+	0.70
						POC	Glx/Cr	18	18	NS	−0.21
Gurler	2021	Sz	3	PRESS	80	Hippocampus	Glx	16	15	NS	0.15^a^
Hjelmervik	2020	Sz	3	PRESS	35	L ST gyrus	Glu	66	60	NR	−0.18
							Gln	66	60	NR	−0.18
							Glx	75	77	NS	−0.03
						R ST gyrus	Glu	33	34	NR	0.47
							Gln	33	34	NR	−0.04
							Glx	35	37	NS	0.30
						ACC	Glu	32	31	NR	0.31
							Gln	32	31	NR	−0.04
							Glx	37	35	NS	0.10
						IF gyrus	Glu	35	34	NR	−0.04
							Gln	35	34	NR	0.24
							Glx	36	38	NS	0.24
Huang	2017	FEP	3	STEAM	9.2	L DLPFC	Glx/Cr	58	43	+	0.41
						R DLPFC		58	43	NR	0.27
Hugdahl	2015	Sz	3	PRESS	35	Temporal cortex	Glx	23	26	–	0.69^a^
						Frontal lobe		23	26	–	0.85
Iwata	2019	uTRS	3	PRESS	35	Caudate	Glu	25	26	NS	−0.07
							Glx	25	26	NS	0.23
						ACC	Glu	26	26	NS	0.63
							Glx	26	26	+	0.70
						DLPFC	Glu	21	26	NS	−0.26
							Glx	21	26	NS	−0.02
		TRS	3	PRESS	35	Caudate	Glu	25	26	NS	0.23
							Glx	25	26	NS	0.22
						ACC	Glu	27	26	NS	0.60
							Glx	27	26	NS	0.67
						DLPFC	Glu	22	26	NS	−0.72
							Glx	22	26	NS	0.06
		Sz	3	PRESS	35	Caudate	Glu	21	26	NS	0.32
							Glx	21	26	NS	0.50
						ACC	Glu	21	26	NS	0.45
							Glx	21	26	NS	0.28
						DLPFC	Glu	21	26	NS	0.55
							Glx	21	26	NS	0.60
Jauhar	2018	FEP	3	PRESS	30	ACC	Glu	28	20	NS	0.11^a^
Jeon	2022	FEP	7	sLASER	70	ACC	Gln	21	10	NR	−0.12
							Glu	21	10	NR	−0.64
Jessen	2013	Sz	3	PRESS	30	ACC	Glx/Cr	18	20	NS	0.00
							Gln/Cr	18	20	NS	0.00
						Frontal lobe	Glx/Cr	18	20	NS	0.00
							Gln/Cr	18	20	NS	0.00
Kaminski	2020	Sz	3	PRESS	80	DLPFC	Glu	55	35	NR	−0.30
Kegeles	2012	Sz	3	PRESS	68	MPFC	Glx	32	22	NR	0.43
						DLPFC		32	22	NR	−0.27
		Sz	3	PRESS	68	MPFC	Glx/Cr	32	22	NR	0.51
						DLPFC		32	22	NR	0.25
Korenic	2020	Sz	3	PRESS	30	ACC	Glu	19	22	NS	−0.08
						Parietal cortex		19	22	NS	−0.32
						Hippocampus		19	22	NS	−0.23
Kraguljac	2019	Sz	3	PRESS	80	ACC	Glx	60	31	NS	0.14
						Hippocampus		58	30	+	−0.56
Kumar	2020	Sz	7	STEAM	17	ACC	Glu	27	45	NR	−0.28
							Gln	27	42	–	−0.53
						Insula	Glu	27	45	NR	−0.08
							Gln	26	44	NR	0.07
						Visual cortex	Glu	26	45	NR	−0.20
							Gln	26	44	NR	0.08
Leptourgos	2022	Sz	3	PRESS	30	ACC	Glx	33	21	NR	−0.24
						Insula		33	21	NR	−0.18
						DLPFC		33	21	NR	−0.36
						Auditory cortex		33	21	NR	−0.07
Li	2020	FEP	3	PRESS	30	ACC	Glu	35	40	NS	0.13
Li	2022	FEP	3	MEGAPRESS	69	MPFC	Glx	32	30	NS	0.32
Lutkenhoff	2010	Sz	3	PRESS	30	MPFC	Glu	9	21	–	−1.07
						Prefrontal WM		9	21	NR	−0.75
						Hippocampus		9	21	NR	−0.49
Marsman	2014	Sz	7	sLASER	28	MPFC	Glu	14	18	NS	−0.14
						POC		15	17	NS	−0.03
Merritt	2019	FEP	3	PRESS	30	ACC	Glu	23	15	NS	0.05
							Glx	23	15	NS	−0.25
						Thalamus	Glu	23	15	NS	−0.06
							Glx	23	15	NS	−0.31
Natsubori	2014	FEP	3	PRESS	15	MPFC	Glx	19	19	NS	0.00
		Sz	3	PRESS	15	MPFC	Glx	25	28	–	−0.62
Nelson	2022	FEP	3	PRESS	80	Hippocampus	Glx	43	37	NS	0.34
Nenadic	2015	FEP	3	PRESS	30	R Hippocampus	Glu	18	42	NR	−0.10
						L Hippocampus	Glu	18	42	NR	0.08
Ongür	2010	Sz	4	MEGAPRESS	68	ACC	Glu/Cr	21	19	NS	0.47
						POC		21	19	NS	−0.04
Ongür	2008	Sz	4	PRESS	30–500	ACC	Glu	17	21	NR	−0.56
							Gln	17	21	NR	0.20
							Glx	17	21	NS	0.56
						POC	Glu	17	21	NR	0.14
							Gln	17	21	NR	0.63
							Glx	17	21	NS	0.76
Onwordi	2021	Sz	3	PRESS	30	ACC	Glu/Cr	18	22	NS	−2.00
							Glx/Cr	18	22	NS	0.47
						Hippocampus	Glu/Cr	17	22	NS	−1.11
							Glx/Cr	17	22	NS	−1.52
Pillinger	2019	TRS	3	PRESS	30	ACC	Glx	19	18	NS	0.40
Plitman	2018	Sz	3	PRESS	35	Striatum	Glu	12	11	NS	−0.35
							Glx	12	11	NS	−0.28
Plitman	2016	FEP	3	PRESS	35	Caudate	Glu	60	60	+	0.62
							Glx	60	60	NS	0.25
Posporelis	2018	FEP	7	STEAM	15	ACC	Glu/Cr	20	20	NS	−0.33
Ragland	2020	Sz	3	PRESS	80	DLPFC	Glu/Cr	38	49	NS	−0.14
Rauchmann	2020	Sz	3	STEAM	20	L Hippocampus	Glx/Cr	38	21	–	0.64^a^
						R Hippocampus		38	21	–	0.59
						DLPFC		39	23	NS	0.29
						Thalamus		39	23	NS	0.22
Reid	2019	FEP	7	STEAM	5	ACC	Glu	21	21	NS	−0.72
							Gln	21	21	–	−0.32
							Glx	21	21	NS	0.00
Reid	2016	Sz	3	PRESS	80	ACC	Glx/Cr	26	18	NS	0.00
						Hippocampus		23	18		0.10
Reid	2013	Sz	3	PRESS	80	Substantia nigra	Glx/Cr	35	22		0.35
Rowland	2013	Sz	3	PRESS	35	ACC	Glx	21	19	NS	−0.57
						CS		21	19	NS	−0.29
Shakory	2018	FEP	3	PRESS	35	Hippocampus	Glu	15	31	NR	−0.19
							Glx	15	31	NR	−0.19
Shirayama	2010	Sz	3	PRESS	30	MPFC	Glu	19	18	NS	−0.13
							Gln	19	18	NS	0.56
Singh	2018	Sz	3	PRESS	33	Hippocampus	Glu/Cr	28	28	–	−0.48
							Glx/Cr	28	28	NS	−0.24
Sivaraman	2018	FEP	3	PRESS	80	Caudate	Glx	14	16	NS	−0.22
Smucny	2022	Sz	3	PRESS	30	DLPFC	Glu	37	42	NS	−0.89
						Visual cortex		36	40	NS	−1.82
		Sz	3	PRESS	30	DLPFC	Glu/Cr	37	42	NS	−1.42
						Visual cortex		36	40	NS	−1.71
Stan	2015	Sz	3	PRESS	70	Hippocampus	Glu	18	16	–	−1.03
Tarumi	2020	Sz	3	PRESS	35	ACC	Glx	26	28	NS	0.71
						Caudate	Glx	27	23	NS	0.06
		TRS	3	PRESS	35	ACC	Glx	25	28	+	0.73
						Caudate	Glx	23	23	NS	0.21
Taylor	2017	Sz	7	STEAM	10	ACC	Glu	16	18	NS	0.56
							Gln	16	18	NS	0.00
						Thalamus	Glu	16	18	NS	0.00
							Gln	16	18	+	1.00
Tayoshi	2009	Sz	3	STEAM	18	ACC	Glu	30	25	NR	−0.46
							Gln	30	25	NR	−0.37
						Basal ganglia	Glu	30	25	NR	0.06
							Gln	30	25	NR	−0.02
Thakkar	2017	Sz	7	MEGA-sLASER	74	OCC	Glu	18	23	–	−0.74
							Gln	18	23	NS	−0.12
							Glx	18	23	NS	−0.68
						R Striatum	Glu	24	20	NS	−0.08
							Gln	23	20	NS	0.14
							Glx	21	20	NS	0.01
						L Striatum	Glu	20	19	NS	0.09
							Gln	19	19	NS	−0.41
							Glx	16	15	NS	−0.21
Théberge	2003	Sz	4	STEAM	20	ACC	Glu	21	21	–	0.71^a^
							Gln	21	21	–	0.72
						Thalamus	Glu	21	21	NS	NA
							Gln	21	21	+	0.87
Tianyi	2017	Sz	3	MEGAPRESS	68	VMPFC	Glu	24	24	+	0.75
							Gln	24	24	–	−1.06
						ACC	Glu	24	24	NS	−0.21
Tunc-Skarka	2009	Sz	3	PRESS	30	Frontal WM	Glu	17	26	NS	0.29
					80			11	21	NS	−0.47
Wang	2019	FEP	7	STEAM	14	ACC	Glu	75	87	–	−0.54
							Gln	74	89	NR	0.06
							Glx	75	88	NR	−0.39
						CS	Glu	68	85	NR	0.17
							Gln	67	83	NR	0.22
							Glx	68	85	NR	0.26
						DLPFC	Glu	72	84	NR	−0.37
							Gln	68	77	NR	0.07
							Glx	71	85	NR	−0.16
						OFC	Glu	61	73	NR	0.16
							Gln	57	52	NR	−0.12
							Glx	62	73	NR	0.18
						Thalamus	Glu	66	74	NR	−0.20
							Gln	52	68	NR	0.35
							Glx	67	74	NR	−0.06
Wang	2016	FEP	3	MEGAPRESS	68	MPFC	Glx	16	23	–	−1.33
Wang	2022	Sz	3	PRESS	30	MPFC	Glx	114	59	NR	−0.44
White	2015	Sz	3	PRESS	80	Substantia nigra	Glx	15	15	+	0.53
Wijtenburg	2021	Sz	7	STEAM	14	ACC	Glu	39	37	–	−0.35
							Gln	39	37	NS	0.22
							Glx	39	37	NS	0.23
						CS	Glu	40	38	NR	0.07
							Gln	39	36	+	0.64
							Glx	39	36	+	0.74
						DLPFC	Glu	38	38	NS	0.22
							Gln	38	36	NS	0.51
							Glx	38	36	NS	0.38
						Hippocampus	Glu	34	31	NS	−0.30
							Gln	29	27	NS	0.14
							Glx	29	27	NS	0.28
						Thalamus	Glu	40	38	NS	−0.06
							Gln	38	38	NS	0.55
							Glx	38	36	NS	0.45
Wijtenburg	2021	Sz	3	PR STEAM	6.5	OCC	Glu	17	18	+	0.76
							Gln	17	18	+	0.78
Wood	2007	Sz	3	PRESS	30	L dACC	Glx	15	14	NS	−0.26
						L rACC		15	14	NS	0.24
						R dACC		15	14	NS	−0.59
						R rACC		15	14	NS	0.11
Yang	2015	FEP	3	MEGAPRESS	69	VMPFC	Glu	22	23	NS	0.39
							Gln	22	23	NS	0.17
Yoon	2020	Sz	3	MEGAPRESS	68	Visual cortex	Glx/Cr	22	30	NS	−0.06

+: higher levels; –: lower levels; ACC: anterior cingulate cortex; Cr: creatine; CT: constant-time; CS: centrum semiovale; dACC: dorsal ACC; DLPFC: dorsolateral prefrontal cortex; FEP: first-episode psychosis; Gln: glutamine; Glu: glutamate; Glx: Glu + Gln; HC: healthy controls; IF: inferior frontal; L: left; MEGA: Mesher-Garwood; MFC: medial frontal cortex; MPF: medial prefrontal region; MPFC: medial prefrontal cortex; MS: milliseconds; OCC: occipital cortex; PFC: prefrontal cortex; POC: parieto-occipital cortex; PRESS: point-resolved spectroscopy sequence; PR: phase rotation; ^1^H-MRS: proton magnetic resonance spectroscopy; R: right; rACC: rostral ACC; Sequence: ^1^H-MRS sequence; sLASER: semi-localization by adiabatic selective refocusing; SPECIAL: spin echo full intensity acquired localized; SSD: schizophrenia spectrum disorders; ST: superior temporal; STEAM: stimulated echo acquisition mode; Sz: schizophrenia; T: field strength in Tesla; TE: echo time; TRS: treatment-resistant schizophrenia; uTRS: ultra-TRS; VMPF: ventromedial prefrontal cortex; WM: white matter.

‘n SDD’ and ‘n HC’ represent maximum number of participants who undertook ^1^H-MRS investigation. Actual sample size may vary depending on SSD, ^1^H-MRS sequence, voxel placement, metabolite and excluded participant data. ‘Result’ shows reported study SSD glutamatergic metabolite level results as statistically significant, represented by higher (+) and lower (–) levels, statistically not significant (NS) or as statistical significance not reported (NR), compared with HC.

a Means and standard deviations were not provided in the study and, as such, the effect size was calculated using t-values instead.

The following descriptions of between-group comparison results denote the direction and magnitude of glutamatergic metabolite effect size (Cohen’s *d*) levels in participants within an SSD group compared with HC, where positive effect sizes indicate higher metabolite levels in SSD groups.

### Qualitative analyses

#### Participant demographics

A total of 2721 HC and 2822 participants with an SSD diagnosis were included in this review, with a mean age ranging from 21 to 66 years across the SSD groups and 21 to 70 years in HC (Table 3). A consistent general trend of higher male participation (i.e. SSD = 67%; HC = 62%) compared with female was observed. Moreover, a clear age progression was noted, from FEP through to uTRS, with participants with more advanced or resistant forms of schizophrenia tending to be older. Overall, there was no significant difference in mean age between SSD participants and HC (*p* = 0.492).

**Table 3.** Participant demographics and clinical characteristics.

	FEP	Sz	TRS	uTRS	HC
*N* (total)	890	1802	93	37	2721
Age (years)	24.20	32.97	40.41	42.10	31.12
Male	608	1233	64	30	1642
Female	308	580	38	7	1013
Medication					
AP-naïve	503	123	–	–	–
AP-free	28	91	–	–	–
AP treatment	189	847	50	–	–
Mixed status	170	732	–	–	–
Clozapine	–	–	43	37	–
CPZ eq.	227.91 (131.46)	438.30 (156.00)	644.69 (229.28)	706.29 (98.33)	–
Measures					
PANSS total	63.82 (17.61)	64.84 (13.91)	77.99 (23.45)	76.74 (9.45)	–
- Positive	22.76 (9.73)	15.21 (3.75)	17.97 (6.74)	19.97 (63.94)	–
- Negative	19.18 (4.64)	16.79 (3.50)	21.80 (6.06)	20.63 (0.42)	–
- General	39.32 (9.22)	31.88 (7.33)	36.97 (10.75)	36.20 (5.14)	–
SANS	18.99 (0.59)	23.81 (16.21)	–	–	–
SAPS	12.41 (0.59)	10.69 (7.45)	–	–	–
BPRS total	48.20 (5.68)	34.26 (14.22)	–	–	–
- Positive	11.85 (2.72)	9.69 (3.02)	–	–	–
- Negative	6.40 (0.77)	6.41 (1.23)	–	–	–

AP: antipsychotics; BPRS: Brief Psychiatric Rating Scale; CPZ eq.: chlorpromazine equivalent doses; FEP: first-episode psychosis; HC: healthy controls; PANSS: Positive and Negative Syndrome Scale; SANS: Scale for the Assessment of Negative Symptoms; SAPS: Scale for the Assessment of Positive Symptoms; SSD: schizophrenia spectrum disorders; TRS: treatment-resistant schizophrenia; uTRS: ultra-TRS.

Age, chlorpromazine equivalent doses and clinical measure scores represent mean averages (mean standard deviations) calculated after using SSD group sample size as a weighing factor. Chlorpromazine equivalent doses in mg.

#### Clinical characteristics

##### Medication status

Clinical participants were described as either being antipsychotic-naïve, antipsychotic-free, being treated with antipsychotics, being treated with clozapine, or mixed (i.e. some participants were medicated, while others were not). FEP studies primarily focused on antipsychotic-naïve individuals (61%), with schizophrenia groups mainly assessing participants medicated with antipsychotics (52%) or mixed medication states (37%). In both TRS and uTRS groups, all participants were treated with antipsychotics (TRS: 60%), particularly clozapine (TRS: 40%; uTRS: 100%). A progression in chlorpromazine equivalent doses was noted, from FEP to uTRS (Table 3).

##### Clinical measures

Overall, followed by the BPRS (26%), the PANSS was the most frequently employed measure (56%) and the only one used among all SSD groups. Both tests captured overall symptom burden and were often accompanied by subscale scores for specific symptom domains (e.g. positive, negative, general). Apart from the SANS and the SAPS, studies did not generally combine different clinical measures within the same participants. Compared with the schizophrenia group, all other SSD group participants tended to have higher positive, negative and general symptom scores (Table 3).

#### FEP-specific characteristics

Thirteen studies employed specific criteria for the duration of psychotic symptoms prior to study inclusion. The average reported range of time from symptom onset to study entry was 31.85 (SD = 17.90) months. Eleven studies reported an average illness duration (i.e. the total time elapsed since symptom onset) of 16.67 (SD = 8.14) months. Sixteen studies reported a mean duration of untreated psychosis (i.e. time elapsed between the first noticeable onset of positive psychotic symptoms and the start of consistent antipsychotic treatment) of 9.45 (SD = 7.53) months.

#### Trends in regional glutamatergic metabolite levels

Overall, approximately 20% of all reported effect size (Cohen’s *d*) findings comparing SSD participants with HC were statistically significant. Statistically significant findings generally exhibited medium to large effect sizes (Cohen’s *d*). Regarding the direction of effect sizes, while Gln and Glx levels tend to be generally higher in SSD groups compared with HC, Glu was generally lower in SSD groups.

**Figure 2.**
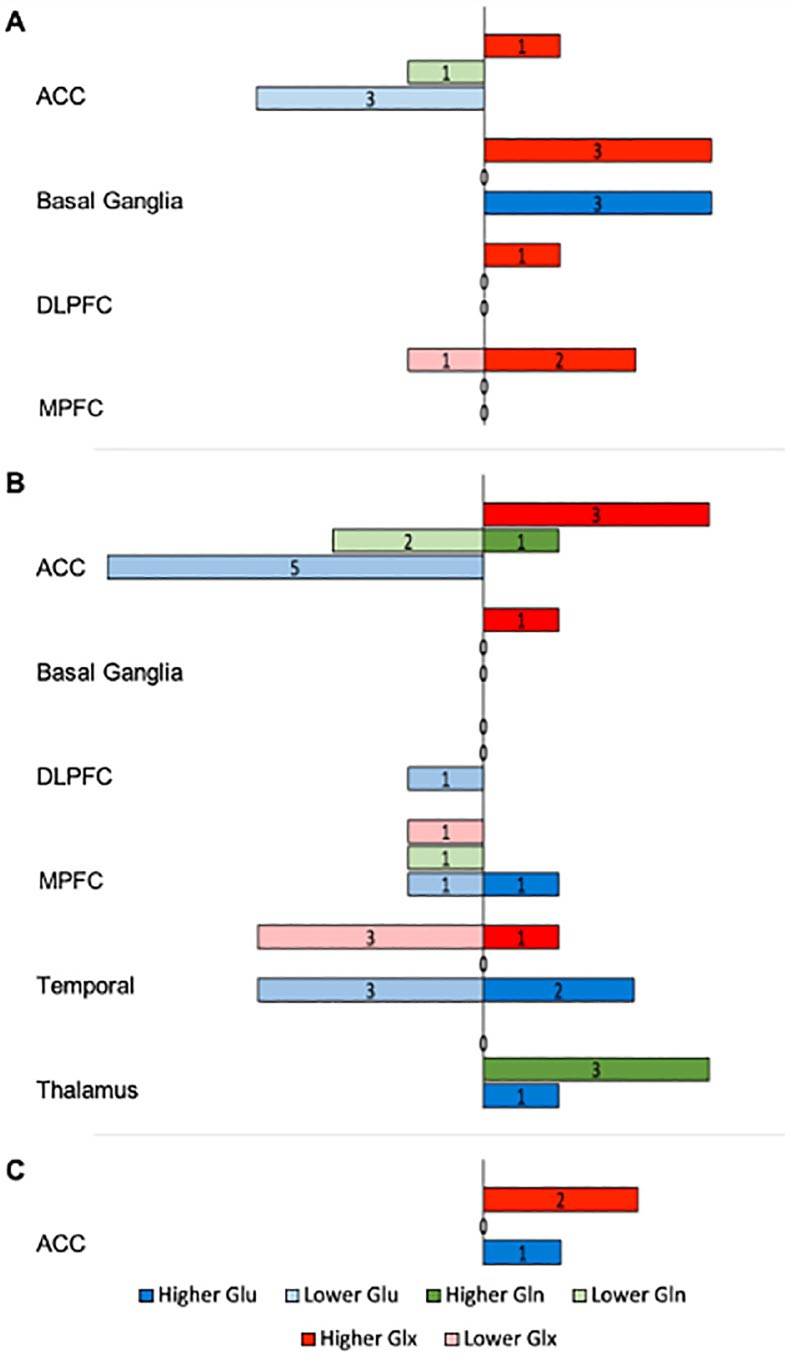
Significant regional glutamatergic metabolite variations between SSD and HC. ACC: anterior cingulate cortex; DLPFC: dorsolateral prefrontal cortex; FEP: first-episode psychosis; Gln: glutamine; Glu: glutamate; Glx: Glu + Gln; MPFC: medial prefrontal cortex; TRS: treatment-resistant schizophrenia; uTRS: ultra-TRS. The figure represents the number of statistically significant studies that reported lower (left) or higher (right) levels of glutamatergic metabolites across brain regions in (A) FEP, (B) schizophrenia and (C) TRS + uTRS, compared with HC; due to small number of studies in TRS and in uTRS, these were combined into a single group (C).

Following are the main trends in effect size magnitude and direction in glutamatergic metabolite levels of SSD groups in relation to HC across brain regions in statistically significant reports (Figure 2). Brain regions with a small number of between-group comparisons (i.e. *n* < 20) were not included in this summary. A detailed account of the findings is provided in Supplemental Materials.

##### Anterior cingulate cortex

The ACC revealed consistent lower levels of Glu for FEP and schizophrenia than HC. Gln appeared lower in schizophrenia. Glx levels were generally higher across all SSD groups.

##### Basal ganglia

Higher levels of Glu and Glx were noted in FEP than HC.

##### Medial prefrontal cortex

Lower levels of Glx were found in schizophrenia than HC.

##### Temporal region

In schizophrenia, while Glu appeared to have both higher and lower levels, Glx levels were generally lower than HC.

##### Thalamus

Findings show consistent higher levels of Gln in schizophrenia than HC.

### Quantitative analysis of effect size

Brain voxels with fewer than 15 between-group comparisons were categorized by their respective brain region (i.e. basal ganglia, frontal lobe, MPF, temporal lobe and visual cortex) for analysis. However, 18 between-group comparisons, corresponding to regions of the centrum semiovale, cerebellar cortex, IFG and insula, were excluded from analysis due to their small number and inability to be categorized by brain region.

#### Comparisons across brain regions for SSD group

**Figure 3.**
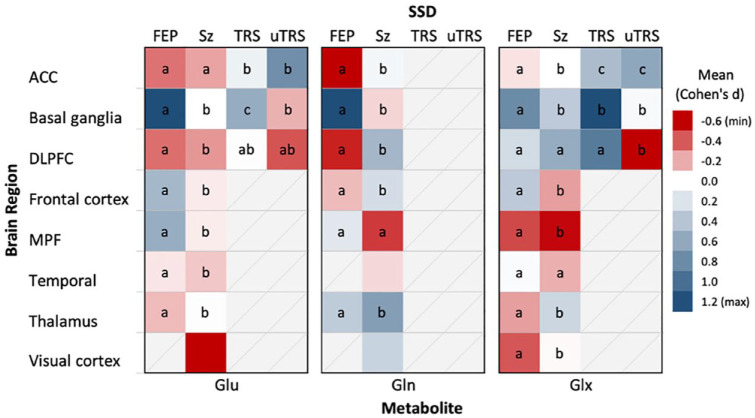
Brain region-specific effect size means of Glu, Gln and Glx in SSDs. ACC: anterior cingulate cortex; DLPFC: dorsolateral prefrontal cortex; FEP: first-episode psychosis; Gln: glutamine; Glu: glutamate; Glx: Glu + Gln; MPF: medial prefrontal; SSD: schizophrenia spectrum disorders; Sz: schizophrenia; TRS: treatment-resistant schizophrenia; uTRS: ultra-TRS. Colours in the heatmap represent weighted Cohen’s d averages, with red indicating negative values and blue positive values. Letters within each cell represent pairwise comparisons of Cohen’s d between SSD groups for each brain region; shared letters indicate no statistically significant difference, while distinct letters indicate significant differences (*p* < 0.05, Bonferroni-corrected).

Figure 3 presents a comparative analysis of SSD groups and HC across various brain regions, focusing on the levels of each glutamatergic metabolite. The strength of the detected effects is represented through a colour gradient, with different shades indicating the magnitude of effect sizes (Cohen’s *d*). Accompanying letters in the figure denote the statistical significance of differences in effect size between SSD groups; groups that share the same letter are not significantly different in their Cohen’s *d* values. Effect sizes (η^2^) for differences between SSD groups and HC ranked Cohen’s *d* values were summarized across brain regions (Table 4).

**Table 4.** Relationship between Cohen’s *d* and SSD group across brain regions.

Brain region	Glu		Gln		Glx	
	η^2^	*p*	η^2^	*p*	η^2^	*p*
ACC	0.095	<0.001	0.052	<0.001	0.111	<0.001
Basal ganglia	0.446	<0.001	0.665	<0.001	0.148	<0.001
DLFPC	0.063	<0.001	0.523	<0.001	0.108	<0.001
Frontal cortex	0.423	<0.001	0.520	<0.001	0.284	<0.001
MPF	0.174	<0.001	0.091	0.014	0.313	<0.001
Temporal	0.003	0.293	–	–	0.004	0.133
Thalamus	0.115	<0.001	0.069	<0.001	0.370	<0.001
Visual cortex	–	–	–	–	0.076	0.006

ACC: anterior cingulate cortex; DLPFC: dorsolateral prefrontal cortex; Gln: glutamine; Glu: glutamate; Glx: Glu + Gln; MPFC: medial prefrontal cortex.

Effect sizes (η^2^) were conducted on ranked Cohen’s *d* values.

##### Glutamate

The basal ganglia (η² = 0.446, *p* < 0.001) and the frontal cortex (η² = 0.423, *p* < 0.001) displayed the greatest Cohen’s d mean differences for Glu, followed by the MPF (η² = 0.174, *p* < 0.001). Notably, while individuals with FEP exhibited moderately higher levels of Glu in the basal ganglia (*d* = 0.576), participants with schizophrenia exhibited moderate decreases in Glu within the visual cortex (*d* = −0.624). Pairwise comparisons found that FEP and schizophrenia consistently differed across brain regions (except ACC, *p* = 1.0, Bonferroni-corrected). In the ACC, the negative Cohen’s *d* in FEP and schizophrenia differed significantly from the positive Cohen’s *d* in TRS and uTRS with a moderate effect (η² = 0.095, *p* < 0.001). Within the DLPFC, all SSD groups revealed consistent negative Cohen’s *d* compared with controls, with a moderate effect (η² = 0.063, *p* < 0.001). A significant difference in Cohen’s *d* between TRS and uTRS was noted only in the basal ganglia (*p* < 0.001).

##### Glutamine

Only FEP and schizophrenia groups were assessed for Gln levels. The analysis revealed strong notable Cohen’s *d* differences in the basal ganglia (η² = 0.665, *p* < 0.001), the DLPFC (η² = 0.523, *p* < 0.001) and frontal cortex (η² = 0.520, *p* < 0.001). Across all regions, except the MPF (*p* = 0.418), pairwise comparisons revealed FEP and schizophrenia differed significantly in Cohen’s *d* (*p* < 0.001, Bonferroni-corrected) and, with the exception of the thalamus, typically differed in direction. Of note, compared with HC, significantly positive large Cohen’s *d* was found in the basal ganglia (*d* = 1.219) for FEP participants compared with controls, and positive medium Cohen’s *d* in the DLPFC (*d* = 0.506) and thalamus (*d* = 0.670) in schizophrenia.

##### Glx

A general trend of positive Cohen’s *d* emerged in SSD groups across brain regions, with TRS notably showing moderate positive Cohen’s *d* in both the basal ganglia (*d* = 0.705) and the DLPFC (*d* = 0.527). uTRS exhibited a negative Cohen’s *d* in the DLPFC (*d* = −0.324), significantly different from the positive Cohen’s *d* of the remaining SSD groups (η² = 0.108; *p* < 0.001). Markedly large Cohen’s *d* differences were found between SSD groups in the thalamus (η² = 0.370, *p* < 0.001), the MPF (η² = 0.313, *p* < 0.001), the frontal cortex (η² = 0.284, *p* < 0.001) and basal ganglia (η² = 0.148, *p* < 0.001). SSD Cohen’s *d* differed significantly across all brain regions (*p* < 0.001), except for the temporal region (η² = 0.004, *p* = 0.133).

#### Glutamatergic levels Cohen’s *d* and clinical variables

##### Medication status

Significant differences were found in glutamatergic metabolite levels across all categories of medication status (*p* < 0.001). Pairwise comparisons revealed significant differences in Cohen’s *d* between all medication statuses (*p* < 0.05, Bonferroni-corrected) in Gln, with some exceptions in Glu and Glx. In Glu, no significant differences in metabolite levels were found between individuals treated with clozapine and those who were antipsychotic-naïve (*p* = 0.589). In Glx, no significant differences were found between individuals treated with mixed antipsychotics and those who were antipsychotic-free (*p* = 1.00), between antipsychotic-free individuals and those treated with clozapine (*p* = 0.442) and between individuals treated with clozapine and antipsychotic-naive participants (*p* = 1.00). Regarding chlorpromazine equivalent doses, though significant Cohen’s *d* correlations were found with both Glu and Gln (*p* < 0.001; Table 5), these were found to be negligible (Glu: *r_s_* = 0.123; Gln: *r_s_* = 0.173).

**Table 5.** Relationship strength between Cohen’s *d* and clinical variables.

	Glu		Gln		Glx	
	*r_s_*	*p*	*r_s_*	*p*	–	*p*
CPZ eq.	0.123	<0.001	0.173	<0.001	−0.046	0.051
PANSS total	0.027	0.323	0.253	<0.001	0.108	<0.001
- Positive	0.205	<0.001	0.013	0.762	0.295	<0.001
- Negative	0.266	<0.001	0.283	<0.001	0.176	<0.001
- General	0.363	<0.001	0.123	0.009	0.294	<0.001
SANS	0.437	<0.001	0.107	0.020	−0.341	<0.001
SAPS	0.461	<0.001	0.013	0.790	−0.298	<0.001
BPRS total	−0.173	<0.001	0.350	<0.001	−0.129	<0.001
- Positive	0.647	<0.001	0.643	<0.001	−0.315	<0.001
- Negative	−0.067	0.210	0.350	<0.001	0.074	0.038

BPRS: Brief Psychiatric Rating Scale; CPZ eq.: chlorpromazine equivalent doses; Gln: glutamine; Glu: glutamate; Glx: Glu + Gln; PANSS: Positive and Negative Syndrome Scale; SANS: Scale for the Assessment of Negative Symptoms; SAPS: Scale for the Assessment of Positive Symptoms.

Monotonic relationships were assessed using the Spearman correlation coefficient (*r_s_*).

##### Clinical measures

Significant correlations were found between glutamatergic metabolite level Cohen’s *d* and most clinical measure scores (*p* < 0.001; Table 5). Particularly, in Glu, strong monotonic relationships were found between Cohen’s *d* and the BPRS positive scale (*r_s_* = 0.647), followed by the SANS (*r*_s_ = 0.437) and the SAPS (*r_s_* = 0.461), and a moderate association with the PANSS general scale (*r_s_* = 0.363). In Gln, the BPRS revealed a strong correlation with the positive scale (*r_s_* = 0.643), followed by a moderate relationship in both the total score (*r_s_* = 0.350) and the negative scale (*r_s_* = 0.350). Notably, Glx exhibited inverse moderate relationships between Cohen’s and the SANS (*r_s_* = −0.341), the SAPS (*r_s_* = −0.298) and the BPRS positive scale (*r_s_* = −0.315). In the PANSS, weak to moderate positive relationships were found in the positive scale (*r_s_* = 0.295) and general scale (*r_s_* = 0.294).

#### Glutamatergic levels Cohen’s *d* and methodological variables

**Figure 4.**
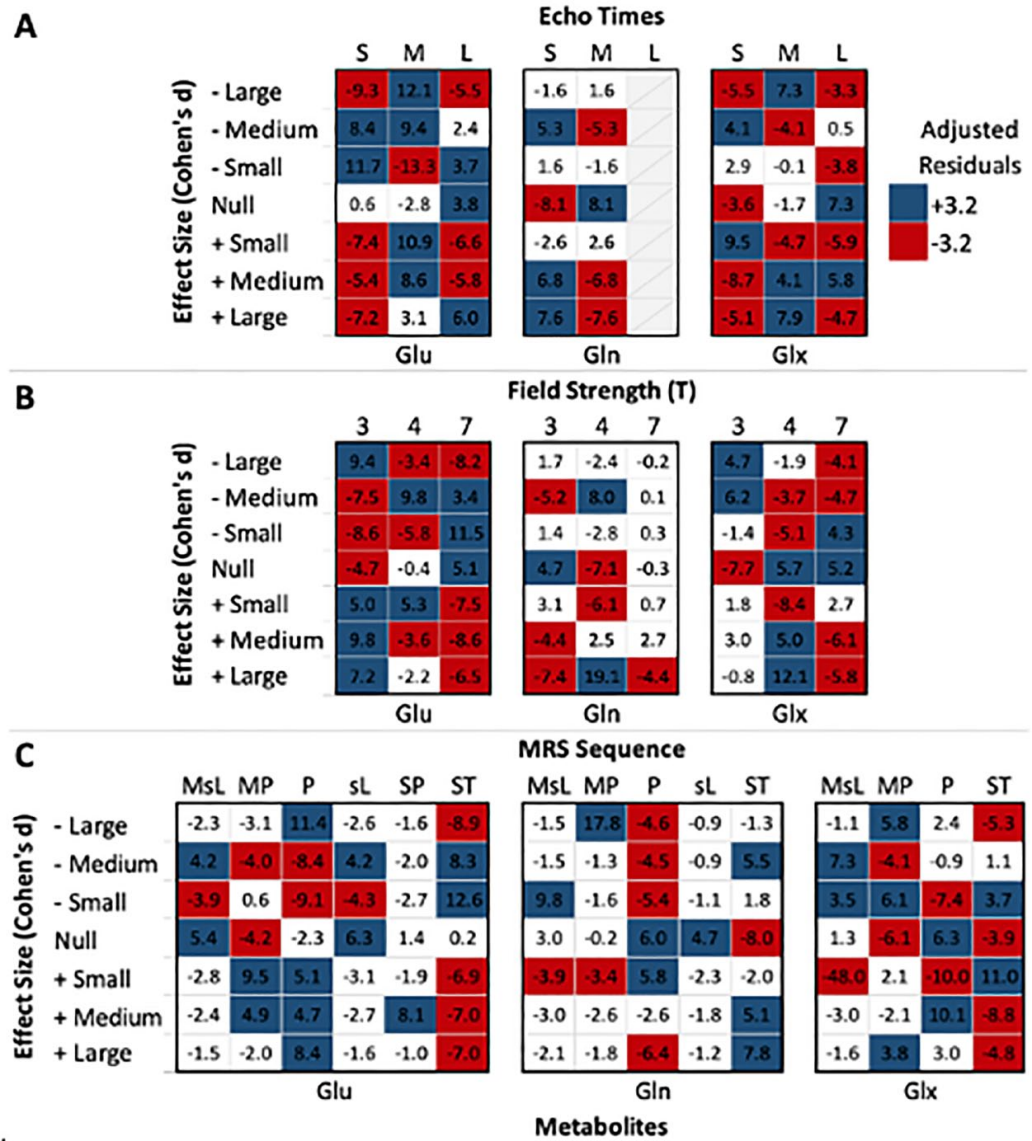
Standardized residuals between Cohen’s *d* and methodological variables. –: negative effect size; +: positive effect size. (A) L: long echo times; M: medium echo times; S: short echo times. (C) MP: MEGA-PRESS; MsL: MEGA-sLASER; P: PRESS; sL: sLASER; SP: SPECIAL; ST: STEAM. The figure depicts the relationship between glutamatergic metabolite Cohen’s *d* and echo time (A), field strength (B) and ^1^H-MRS methodology (C). The values within each cell represent the adjusted standardized residuals for each variable combination. Cells shaded in red represent adjusted standardized residuals below −3.2, while cells shaded in blue represent adjusted standardized residuals above 3.2.

Statistically significant associations were found between Cohen’s *d* for all three glutamatergic metabolites and echo time, field strength and MRS sequence (Table 6). Figure 4 depicts the adjusted standardized residuals across these associations, highlighting areas of significant deviation from the expected frequencies under the assumption of independence.

**Table 6.** Association strength between Cohen’s *d* and methodological variables.

	Glu		Gln		Glx	
	*V*	*p*	*V*	*p*	*V*	*p*
Echo times	0.443	<0.001	0.346	<0.001	0.312	<0.001
Field strength	0.287	<0.001	0.427	<0.001	0.239	<0.001
MRS sequence	0.219	<0.001	0.332	<0.001	0.208	<0.001

Gln: glutamine; Glu: glutamate; Glx: Glu + Gln.

The strength of association was calculated using Cramer’s V (*V*).

##### Glutamate

Analyses revealed a moderate to possibly strong association between Glu levels Cohen’s *d* and echo time (Table 6; χ^2^ = 624.08, *df* = 12, *p* < 0.001, *V* = 0.313), field strength (χ^2^ = 529.31, *df* = 12, *p* < 0.001, *V* = 0.287) and MRS sequence (χ^2^ = 771.89, *df* = 30, *p* < 0.001, *V* = 0.219). Notably, while short echo times yielded fewer than expected positive Cohen’s *d* (Figure 4), medium echo times showed a greater number of both negative and positive Cohen’s *d*. Regarding field strength, while scans at 3 T appeared to yield more positive Cohen’s *d* than expected, the opposite was noted at 7 T. As for MRS sequence, overall positive Cohen’s *d* were more frequent with MEGAPRESS and PRESS, as opposed to less frequent with STEAM.

##### Glutamine

In Gln, these associations were even more pronounced, with echo time (χ^2^ = 165.21, *df* = 6, *p* < .001, *V* = 0.346), field strength (χ^2^ = 516.18, *df* = 12, *p* < 0.001, *V* = 0.427) and MRS sequence (χ^2^ = 625.11, *df* = 24, *p* < 0.001, *V* = 0.332) displaying robust connections with Cohen’s *d* (Table 6). Overall, Gln had the fewest cells with adjusted residuals that deviated from the expected (Figure 4).

##### Glx

Cohen’s *d* in Glx had a moderate to possibly strong association with methodological variables (echo time: χ^2^ = 339.26, *df* = 12, *p* < 0.001, *V* = .221; field strength: χ^2^ = 401.61, *df* = 12, *p* < 0.001, *V* = 0.239; MRS sequence: χ^2^ = 457.80, *df* = 18, *p* < 0.001, *V* = .208). Of note, field strengths of 3 T and MEGA-sLASER MRS sequences had the fewest deviations from expected.

## Discussion

Using the largest pooled dataset to date, this review presents a comprehensive examination of the variations in glutamatergic metabolite levels across different SSD groups and brain regions, as well as across clinical and methodological variables. Given the large and diverse datasets in this field, organizing the data into coherent themes or patterns poses significant challenges. By leveraging an integrated approach that synthesizes both qualitative observations and quantitative analyses, this review provides a more robust understanding of the intricacies underlying these variations.

### Regional glutamatergic metabolite differences across SSD groups

#### Qualitative trends

Approximately 20% (*n* = 57) of comparisons revealed significant differences in glutamatergic metabolite levels between SSD groups and HC, generally with medium to large effect sizes. Overall, compared with HC, most studies reporting significant metabolite levels found higher Gln (60%) and Glx (70%) levels in SSD groups, while Glu (58%) was generally reported lower in SSD groups. The overall body of evidence, however, remains mixed, with no uniform pattern across SSD groups or brain regions (Figure 2). Noteworthy trends were highlighted in the metabolite measurements across brain regions and SSD groups. Specifically, compared with HC, most studies reporting significance in the ACC exhibited lower Glu levels in FEP and schizophrenia and higher Glx levels throughout SSD groups. Studies reporting significance in the basal ganglia mainly showed elevated Glu and Glx levels in FEP, suggesting the possibility of distinct regional variations in glutamatergic metabolite alterations early in the disease progression. In the thalamus, studies suggest higher levels of Gln in schizophrenia groups, which is consistent with previous meta-analyses (Merritt et al., 2016; Salavati et al., 2015).

#### Quantitative insights

Our findings reveal significant variability in the distribution of glutamatergic metabolite concentrations across SSD groups or brain regions, suggesting a complex pathology that varies with disease state and neuroanatomy, as well as varying patterns of glutamatergic metabolite alterations across SSD groups and different brain regions. However, for Glu, schizophrenia revealed consistently lower Cohen’s *d* values across all brain regions, except for the basal ganglia (*d* = 0.003), supporting the hypothesis of lower overall Glu levels across the brain in this SSD group, compared with HC.

Significant differences in Cohen’s *d* between SSD groups and HC were found across all brain regions, with the exception of the temporal region. Compared with HC, the basal ganglia exhibited the largest Cohen’s *d* mean difference for Gln in the FEP group, and the most statistically significant Gln differences between both FEP and schizophrenia groups. These significant differences in Cohen’s *d* between FEP and schizophrenia were maintained throughout most brain regions and glutamatergic metabolites (Figure 3), highlighting an early hyperglutamatergic state, which may normalize or invert as the illness progresses or with treatment, or potentially reflect different stages of glutamatergic dysfunction. These findings are consistent with previous meta-analyses which highlighted higher levels of Glu and Glx in the basal ganglia of patients with FEP and schizophrenia (Merritt et al., 2016; Nakahara et al., 2022; Poels et al., 2014). This evidence joins previous work in supporting a glutamatergic dysfunction in the basal ganglia which is apparent at both early stages and throughout illness progression. Table 7 provides an updated summary of these findings.

**Table 7.** Summary of ^1^H-MRS glutamatergic metabolite level findings.

SSD	Glu	Gln	Glx
FEP	↑ **BG**, FC, MPF ↓ ACC, DLPFC	↑ **BG**, Thal ↓ ACC, DLPFC	↑ BG, FC ↓ MPF, VC
Schizophrenia	↓ ACC, DLPFC, **VC**	↑ **DLPFC**, FC, **Thal**, VC ↓ MPF	↑ BG, DLPFC ↓ MFC
TRS	↑ BG	–	↑ ACC, **BG**, **DLPFC**
uTRS	↑ ACC ↓ DLPFC	–	↑ ACC ↓ DLPFC

↑: higher levels; ↓: lower levels; ACC: anterior cingulate cortex; BG: basal ganglia; DLPFC: dorsolateral prefrontal cortex; FEP: first-episode psychosis; Gln: glutamine; Glu: glutamate; Glx: Glu + Gln; ^1^H-MRS: proton magnetic resonance spectroscopy; MPF: medial prefrontal region; SSD: schizophrenia spectrum disorders; Thal: thalamus; TRS: treatment-resistant schizophrenia; uTRS: ultra-TRS.

The table provides a summary of the findings in this review regarding glutamatergic metabolite levels across SSD groups and brain regions, compared with HC; in **bold** are brain regions where effect sizes (Cohen’s *d*) are moderate and large in strength.

These findings, however, must be considered in the broader context of participant medication status, as well as methodological factors, as these may influence the measurement of glutamatergic metabolite concentrations. In addition, the predominance of male participants (SSD = 67%) highlights a potential gender bias into the findings. Sex-specific factors, such as hormonal differences and variations in drug metabolism (Brand et al., 2022), may affect glutamatergic system modulation differently in males and females.

#### Medication status

Most SSD participants (74%) were being treated with antipsychotic medication at the time of their enrolment, which could influence the measurement of glutamatergic metabolite concentrations in the brain (Egerton et al., 2017, 2021; Van der Heijden et al., 2004). Significant variations in Glu, Gln and Glx levels across different medication statuses suggest that these metabolites may be particularly sensitive to antipsychotic medication effects and serve as potential pharmacokinetic biomarkers. The lack of significant differences in Glu levels between clozapine-treated and antipsychotic-naïve participants, alongside the negligible correlations with chlorpromazine equivalent doses, contributes to the understanding that clozapine treatment may contribute to Glu modulation (Fukuyama et al., 2019; Hribkova et al., 2022; McQueen et al., 2021). While antipsychotic-naïve FEP individuals exhibit distinct glutamatergic profiles, the progression to higher chlorpromazine equivalent doses in uTRS does not appear to linearly correlate with Glu or Gln levels, suggesting that the effects of antipsychotics on glutamatergic metabolites are not straightforward and may be influenced by long-term antipsychotic treatment, illness progression, or individual patient factors. Future research should aim to elucidate the effects of individual antipsychotic drugs and dosages on neurometabolite levels.

#### Clinical measures

This review found significant relationships between Cohen’s *d* for glutamatergic metabolite levels and clinical measures, underscoring the potential relevance of these metabolites in the pathophysiology and symptomatology of schizophrenia. Regarding symptom scores, non-schizophrenia groups tend to have higher scores on positive, negative and general symptoms, suggesting that the severity and presentation of symptoms may evolve with illness progression and treatment history. Moderate and strong monotonic relationships between both Glu and Gln and most clinical measures, mainly the BPRS, SANS and SAPS, suggest a significant association between glutamatergic dysfunction and psychotic symptoms.

#### Methodological considerations

The statistically significant associations between Cohen’s *d* for glutamatergic metabolites echo time, field strength and ^1^H-MRS sequence highlight the importance of considering methodological factors in neuroimaging studies. These moderate to strong associations can considerably impact the MRS measurement of metabolite concentrations.

The association between glutamatergic metabolite levels and echo time may reflect the sensitivity of metabolite measurements to T2 relaxation effects, which can vary with echo time. While shorter echo times can minimize J-evolution and T2 relaxation, longer echo times can decrease macromolecule contributions and improve the separation of Glu and Gln peaks (Wong et al., 2018). Notably, the results suggest that shorter and longer echo times yield fewer positive Cohen’s *d* values for Glu, while negative values are more prevalent at short and medium echo times.

Field strength also appears to influence the magnitude of the observed effects. Though higher field strengths amplify proton polarization and signal intensity, resulting in improved spectral resolution and clearer separation of metabolite signals from noise (Snyder and Wilman, 2010), only in Gln were there few deviations from the expected at 7 T. In Glu, field strengths of 7 T yielded fewer positive Cohen’s *d* values and more negative values, the opposite of 3 T. Researchers conducting ^1^H-MRS investigations should carefully consider the potential impact of field strength selection on their findings, particularly related to Glu and Glx measurements.

Furthermore, specific sequences like MEGAPRESS and PRESS, as opposed to STEAM, tended to yield more positive Cohen’s *d* values for Glu, implying that certain ^1^H-MRS sequences could be more reliable for detecting changes in glutamatergic metabolites. These findings warrant further investigation to understand the underlying mechanisms by which different ^1^H-MRS sequences influence the detection of glutamatergic metabolites.

Future investigations into the influence of methodological factors on glutamatergic metabolite measurements should explore the interaction and relationship between these factors and neurometabolite quantification. In addition, the regional heterogeneity of the brain and its potential impact on these measurements should be examined.

### Strengths, limitations and future directions

A significant strength of this review is the incorporation of a large number of studies and comparisons, which enhances the statistical power and generalizability of the findings. The combination of qualitative and quantitative approaches allowed for a comprehensive assessment of the data, capturing both the magnitude of effects and underlying patterns. Compared with earlier reviews, ours extends up to June 2022, encompassing recent studies not included in previous meta-analyses, the latest of which only covered literature up to November 2020 (Nakahara et al., 2022), and an additional 447 SSD participants. Furthermore, weighting of Cohen’s *d* averages based on SSD group participant sample size further improved the precision of the effect size estimates and ensured that studies with larger sample sizes, which provide more reliable estimates of the true effect, have a proportional influence on the overall analysis. In addition, the addition of eta-square values and Cramer’s V provided a more thorough analysis in considering the magnitude and relevance of the detected effects.

However, the main limitation of this review is the limited number of available studies, particularly those examining Gln, TRS and uTRS. Also, several studies investigating FEP fail to consider symptom onset and lack follow-up evaluations to track illness progression. Thus, conclusions drawn regarding FEP might not accurately depict its role in the progression towards schizophrenia, given that FEP can either remain stable, transition into schizophrenia or develop into other disorders. Moreover, the heterogeneity in brain voxel placement and size, ^1^H-MRS methodology and scanner specifications hinder the ability to draw significant statistical relationships between studies due to the lack of direct comparability among these variables. In addition, while the review attempted to control for medication status, the complexity of antipsychotic treatment regimens, polypharmacy, symptomatic presentation and their impact on glutamatergic metabolism could not be fully disentangled. This variability underscores the need for future research employing standardized methodologies, larger sample sizes and comprehensive assessment of clinical heterogeneity.

To better understand the involvement of glutamatergic metabolites in the pathophysiology of schizophrenia in future research, we propose the following: (1) Focus on brain regions consistently showing changes in glutamatergic metabolite levels, such as the ACC, basal ganglia and DLPFC in FEP, the ACC and thalamus in schizophrenia and the ACC in TRS and uTRS; (2) adopt an operational definition of FEP with a focus on the onset of initial psychotic symptoms; (3) conduct follow-up diagnostic assessments for FEP to monitor progression into schizophrenia, distinguishing early stages of schizophrenia from other conditions; (4) increase the number of TRS and uTRS studies; (5) assess Glu and Gln metabolite levels individually rather than the combined signals (i.e. Glx) for better differentiation; (6) opt for a field strength of 3 T or above to avoid Glu signal contamination with Gln; (7) increase statistical power with larger sample sizes and (8) employ greater efforts in reducing recruitment gender bias. These recommendations stem from the evidence presented in this review, which provides support for the glutamate hypothesis of schizophrenia by presenting evidence of consistent abnormal levels of glutamatergic metabolites throughout the course of SSD, particularly in the basal ganglia, DLPFC, thalamus and visual cortex. Nonetheless, further research is needed to fully elucidate the precise mechanisms underlying the relationship between glutamate abnormalities and the emergence and manifestation of symptoms throughout the course of the SSD.

## Conclusion

The results of this comprehensive review emphasize evidence of altered levels of glutamatergic metabolites across the SSD, with a particular focus on certain areas of the brain. Given the considerable heterogeneity in the literature, we highlight the importance of standardized future research for the assessment of glutamatergic metabolites using ^1^H-MRS.

## Supplemental Material

sj-docx-1-anp-10.1177_00048674241254216 – Supplemental material for Glutamatergic neurotransmission in schizophrenia: A systematic review and quantitative synthesis of proton magnetic resonance spectroscopy studies across schizophrenia spectrum disorders Supplemental material, sj-docx-1-anp-10.1177_00048674241254216 for Glutamatergic neurotransmission in schizophrenia: A systematic review and quantitative synthesis of proton magnetic resonance spectroscopy studies across schizophrenia spectrum disorders by Jamie J Lopes, Sean P Carruthers, Denny Meyer, Brian Dean and Susan L Rossell in Australian & New Zealand Journal of Psychiatry
