# Supplementary material for: Glutamatergic neurotransmission in schizophrenia: A systematic review and quantitative synthesis of proton magnetic resonance spectroscopy studies across schizophrenia spectrum disorders
Source: Aust N Z J Psychiatry. 2024 May 29;58(11):930–51. doi: 10.1177/00048674241254216 (PMC11529133; doi:10.1177/00048674241254216)
Supplement: sj-docx-1-anp-10.1177_00048674241254216 – Supplemental material for Glutamatergic neurotransmission in schizophrenia: A systematic review and quantitative synthesis of proton magnetic resonance spectroscopy studies across schizophrenia spectrum disorders [file sj-docx-1-anp-10.1177_00048674241254216.docx]

**SUPPLEMENTAL MATERIALS**

**Title**

Glutamatergic neurotransmission in schizophrenia: a systematic review and quantitative synthesis of ^1^H-MRS studies across schizophrenia spectrum disorders.

**Authors**

Jamie J. Lopes (JJL)^a^, Sean P. Carruthers (SPC)^a^, Denny Meyer (DM)^a^, Brian Dean (BD)^a,b^, Susan L. Rossell (SLR)^a,c^

**Affiliations**

^a^ Centre for Mental Health, Swinburne University of Technology, Melbourne, VIC, 3122, Australia.

^b^ Molecular Psychiatry Laboratory, The Florey Institute of Neuroscience and Mental Health, Melbourne, VIC, 3065, Australia.

^c^ Psychiatry, St Vincent’s Hospital, Melbourne, VIC, 3065, Australia.

**Statistical Significance and Effect Size**

Overall, approximately 20% of all reported findings were statistically significant. Statistically significant findings generally exhibited medium to large effect sizes. Regarding direction of effect sizes, while Gln and Glx levels tend to be generally higher in SSD subgroups compared to healthy controls, Glu was generally lower in SSD subgroups.

Overall, the majority of between-group comparisons showed a null effect size (n = 98), followed by 95 with a small effect, 71 with a medium effect, and 39 with a large effect (Figure 1).

**Supplemental Figure 1**

*Frequency Count of Effect Size Among Statistical Significance Groups*

*
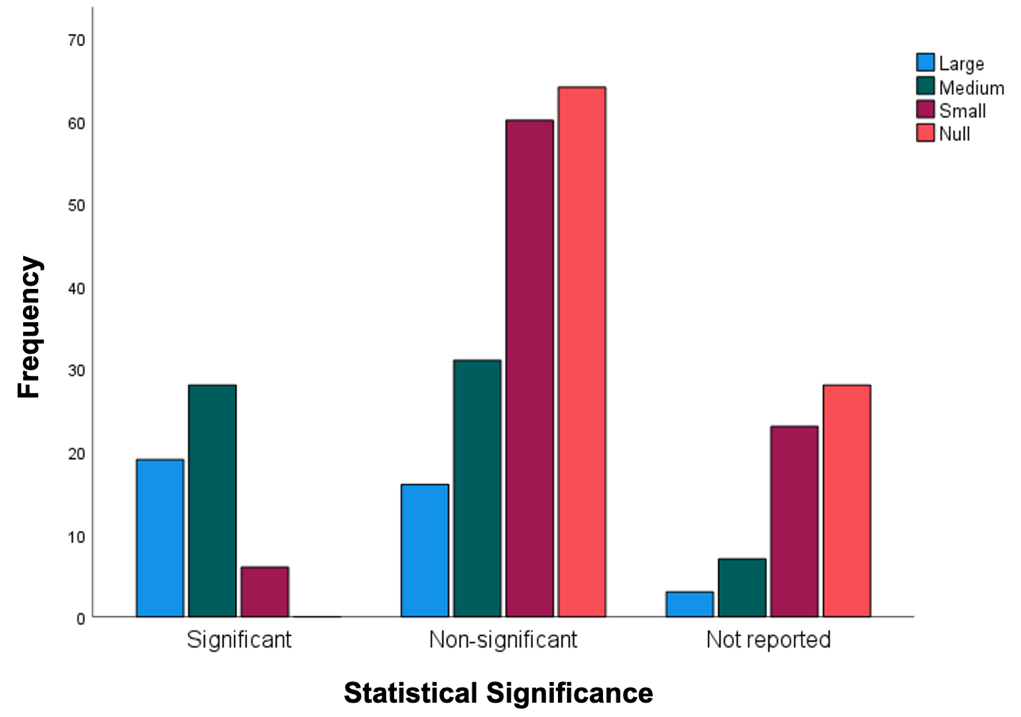
*

Note: Effect sizes were calculated using Cohen’s d, where “Null”, “Small”, “Medium”, and “Large” effect sizes were considered standardised mean differences of d < 0.2, d = 0.2 to 0.5, d = 0.5 to 0.8, and d > 0.8, respectively. “Statistical significance” as reported in the original manuscripts at α = 0.05.

***Group Comparison Across Glutamatergic Metabolites***

Most of the between-group comparisons assessed levels of Glx (n = 132), closely followed by Glu (n = 125), and relatively few assessed Gln levels (n = 47). Table 1 summarises the number of between-group comparisons investigating glutamatergic metabolite levels in clinical subgroups compared to healthy controls.

**Supplemental Table 1**

*Glutamatergic Metabolite Level Differences*

| Metabolite | Comparisons | Significant | Non-Significant | Not Reported | ↑ | ↓ |
| --- | --- | --- | --- | --- | --- | --- |
| Glu | 125 | 24 | 69 | 32 | 50 (10) | 74 (14) |
| Gln | 47 | 10 | 21 | 16 | 30 (6) | 17 (4) |
| Glx | 132 | 23 | 87 | 22 | 81 (16) | 51 (7) |

Note: The table represents the number of between-group comparisons (i.e., Comparisons) reporting on glutamatergic metabolite level differences in clinical subgroups compared to healthy controls. Values in parenthesis indicate the number of statistically significant (i.e., Significant) comparisons in that category. One between-group comparison did not provide t-values in the respective study and, as such, metabolite direction could not be determined. Statistical significance considered at α = 0.05.

Abbreviations: ↑, higher levels; ↓, lower levels; Gln, glutamine; Glu, glutamate; Glx, glutamate+glutamine.

**Glu.** Most of the statistically significant results had either a large (n = 8) or a medium effect size (n = 13; Figure 2) and tend to report on clinical subgroups having lower metabolite levels compared to healthy controls (n = 14).

**Gln.** Most of the statistically significant between-group comparisons appear to show large (n = 5) and medium (n = 4) effect sizes (Figure 2), with a trend toward higher levels (n = 6) in clinical subgroups compared to healthy controls.

**Glx.** Most of the statistically significant between-group comparisons report on large (n = 7) and medium (n = 14) effect sizes, with clinical subgroups tending toward higher levels of the metabolite (n = 16).

**Supplemental Figure 2**

*Effect Size Among Glu, Gln and Glx and Statistical Significance*


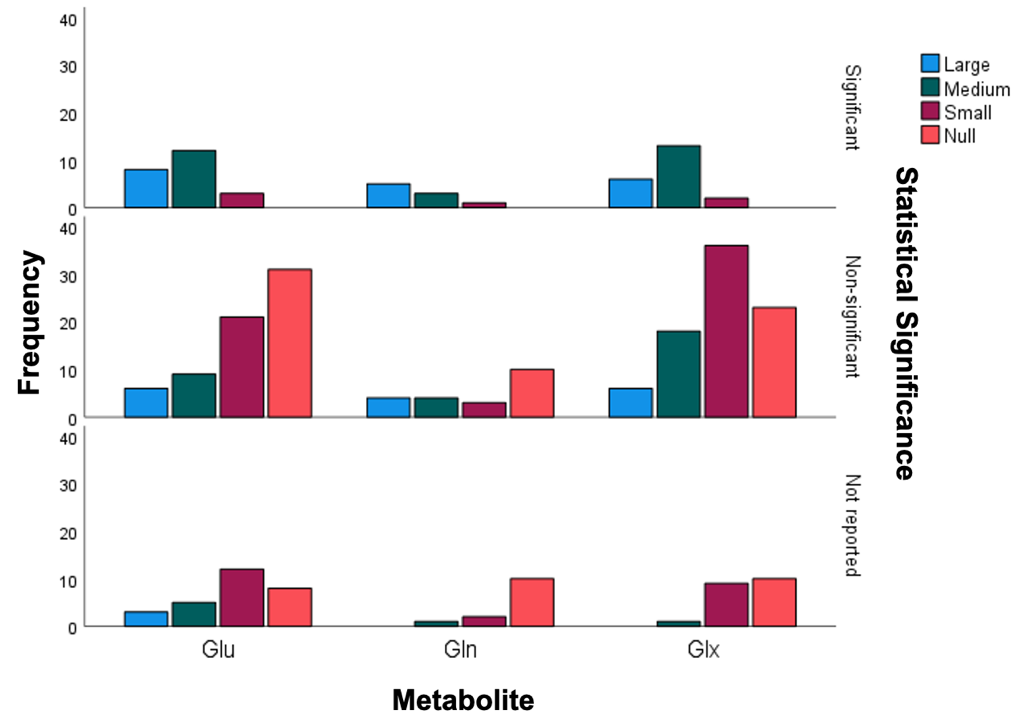


Note: Effect sizes were calculated using Cohen’s d, where “Null”, “Small”, “Medium”, and “Large” effect sizes were considered standardised mean differences of d < 0.2, d = 0.2 to 0.5, d = 0.5 to 0.8, and d >0.8, respectively. “Statistical significance” as reported in the original manuscripts at α = 0.05.

Abbreviations: Gln, glutamine; Glu, glutamate; Glx, Glu+Gln.

***Group Comparison Across SSD Groups***

The following Table 2 presents a summary of the results from between-group comparisons investigating glutamatergic metabolite levels in clinical subgroup cohorts compared to healthy controls. The majority of statistically significant findings had a medium or large effect size (Figure 3). Given the small number of studies for both TRS (n = 5) and uTRS (n = 2) groups, these were pooled and considered jointly in the following analyses.

**Supplemental Table 2**

*Glutamatergic Metabolite Level Differences Across SSD groups*

| SSD | Significant (+/-) | Not Significant (+/-) |
| --- | --- | --- |
| FEP | Glu: 4 / 3  Gln: - / 1  Glx: 7 / 1 | NS: 20 / 11  NR: 11 /11 |
| Schizophrenia | Glu: 5 / 11  Gln: 6 / 3  Glx: 7 / 6 | NS: 65 / 60  NR: 19 / 23 |
| TRS + uTRS | Glu: 1 / -  Glx: 2 / - | NS: 12 / 8  NR: 2 / 4 |

The table represents the number of between-group comparisons reporting on glutamatergic metabolite level differences in SSD groups compared to healthy controls.

Note: t-values for one between-group comparison were not provided in the respective study and, as such, metabolite direction could not be determined. Statistical significance (i.e., Significant) considered at α = 0.05.

Abbreviations: +/-, higher levels/lower levels; FEP, first-episode psychosis; Gln, glutamine; Glu, glutamate; Glx, glutamate+glutamine; NR, statistical significance not reported; NS, statistically not significant; SSD, schizophrenia spectrum disorder; TRS, treatment-resistant schizophrenia; uTRS, ultra-treatment-resistant schizophrenia.

**FEP.** Statistical significance was found in 16 between-group comparisons (Table 2), with most revealing a medium (n = 7) or large effect size (n = 8), and one having a small effect size (Figure 3). Statistical significance was either not found or not reported in 53 between-group comparisons.

**Schizophrenia.** Thirty-eight between-group comparisons reported statistically significant results (Table 4), with over half (n = 22) of the comparisons revealing a medium effect size (Figure 3). Statistical significance was either not found or not reported in 168 between-group comparisons.

**TRS+uTRS.** Three between-group comparisons found statistically significant higher metabolite levels (Table 2), two of which had a medium effect size for Glx in both TRS and uTRS and one had a large effect for Glu in TRS (Figure 3). Statistical significance was either not found or not reported in 26 between-group comparisons.

**Supplemental Figure 3**

*Effect Size Among Clinical Subgroups and Statistical Significance*


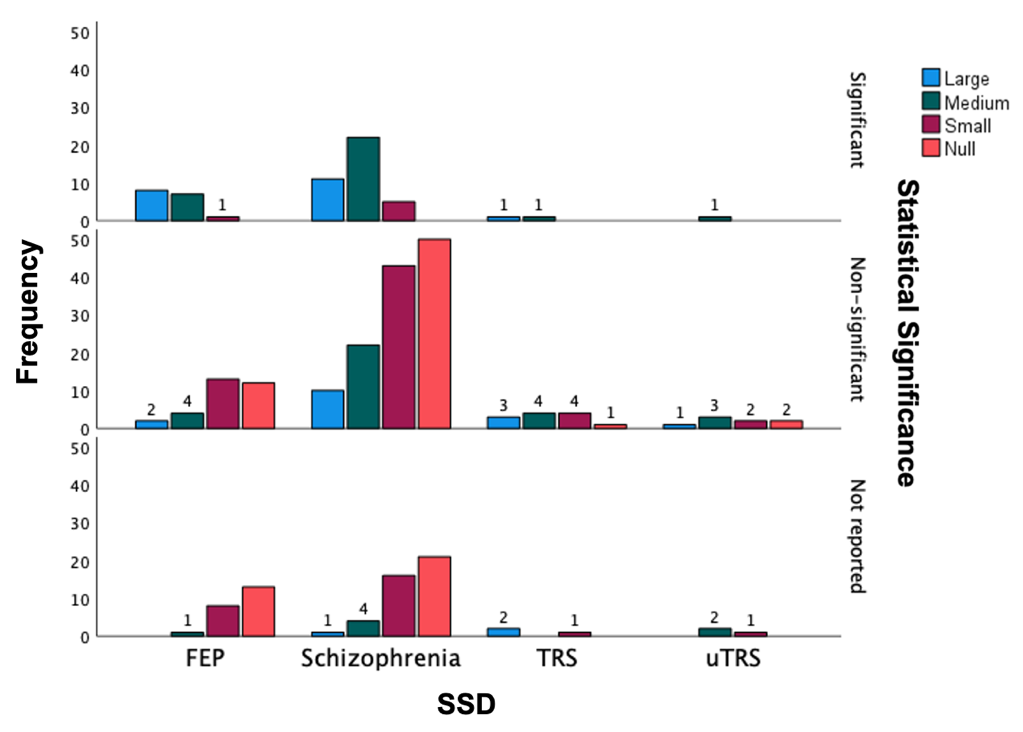


Note: Effect sizes were calculated using Cohen’s d, where “Null”, “Small”, “Medium”, and “Large” effect sizes were considered standardised mean differences of d < 0.2, d = 0.2 to 0.5, d = 0.5 to 0.8, and d >0.8, respectively. “Statistical significance” as reported in the original manuscripts at α = 0.05.

Abbreviations: FEP, first-episode psychosis; SSD, schizophrenia spectrum disorders; TRS, treatment-resistant schizophrenia; uTRS, ultra-TRS.

**Group Comparison Across Brain Regions**

***ACC***

Forty-seven ^1^H-MRS studies investigated glutamatergic metabolites in the ACC. Of these, 34 studies reported on Glu, 17 on Gln and 29 on Glx, from which 21, 8, and 22 studies, respectively, reported non-significant changes and 17 reported significant changes in glutamatergic metabolite levels (table 2). Compared to healthy controls, overall patient clinical subgroups reported significantly higher levels in one study of Glu (Demjaha et al., 2014), one study of Gln (Bustillo et al., 2014), and in six Glx studies (Bustillo et al., 2014; Bustillo et al., 2010; Cadena et al., 2018; Chiu et al., 2018; Iwata et al., 2019; Tarumi et al., 2020). Significantly lower results were found in nine studies reporting on Glu (Chiappelli et al., 2018; Gallinat et al., 2016; Godlewska et al., 2021; Reid et al., 2019; Tayoshi et al., 2009; Théberge et al., 2003; Wang et al., 2019; Wijtenburg et al., 2021a; Falkenberg et al., 2014), and in three studies investigating Gln (Godlewska et al., 2021; Théberge et al., 2003; Kumar et al., 2020). Absolute effect sizes values range from d = 3.85 to d = 0.35, with the majority having a medium effect size.

For Glu, higher levels were reported in one TRS study (Demjaha et al., 2014), whilst lower levels were reported in three FEP studies (Godlewska et al., 2021; Reid et al., 2019; Wang et al., 2019) and in six schizophrenia studies (Chiappelli et al., 2018; Gallinat et al., 2016; Tayoshi et al., 2009; Théberge et al., 2003; Wijtenburg et al., 2021a; Falkenberg et al., 2014). Higher leves in Gln were reported in one schizophrenia study (Bustillo et al., 2014), and lower levels in the metabolite were reported in one FEP between-groups comparison (Godlewska et al., 2021) and in two schizophrenia studies (Théberge et al., 2003; Kumar et al., 2020). Higher levels in Glx were reported in one FEP study (Chiu et al., 2018), three schizophrenia studies (Bustillo et al., 2014; Bustillo et al., 2010; Cadena et al., 2018), in one TRS study (Tarumi et al., 2020), and in one uTRS study (Iwata et al., 2019). No lower levels were reported in Glx levels across clinical subgroups.

***MPFC***

Fifteen studies assessed glutamatergic metabolite levels in the MPFC of patients with FEP and schizophrenia, seven of which reported significant findings. Overall, significantly elevated levels were reported in one study for Glu (Tianyi et al., 2017) and in two Glx studies (de la Fuente-Sandoval et al., 2018; Kegeles et al., 2012). Significantly lower levels were reported across all glutamatergic metabolites, with one study reporting these findings for Glu (Lutkenhoff et al., 2010), one for Gln (Tianyi et al., 2017), and three studies for Glx (Natsubori et al., 2014; Wang et al., 2016; Wang et al., 2022). Absolute effect sizes from statistically significant findings range from d= 0.62 to d = 1.33.

In FEP, only Glx showed significant findings with higher levels reported in one study (de la Fuente-Sandoval et al., 2018) and lower levels reported in another (Wang et al., 2016). In schizophrenia, while higher levels were reported in one Glu study (Tianyi et al., 2017) and in one Glx study (Kegeles et al., 2012), lower levels were described in one Glu study (Lutkenhoff et al., 2010), one Gln study (Tianyi et al., 2017), and in two Glx studies (Natsubori et al., 2014; Wang et al., 2022).

***Frontal Region***

**DLPFC.** Of the fourteen studies reporting on the DFLPC, three reported significant results with medium and small effect sizes: one on the higher levels of Glx in patients with FEP (Huang et al., 2017) and two on the lower levels of Glu in schizophrenia (Corcoran et al., 2020; Kaminski et al., 2020).

**Others.** Three studies investigated glutamatergic metabolites in the frontal lobe, four in the frontal white matter, and one in the IFG. From these, one study reported statistically significant findings, with Glx levels found to be lower in the frontal lobe of patients with schizophrenia with a medium-to-large effect size (Hugdahl et al., 2015).

***Basal Ganglia***

While fifteen studies assessed glutamatergic levels in the left basal ganglia, caudate, putamen, substantia nigra, striatum, and associative striatum, six of these reported significant findings, all of which with medium and large effect sizes. Overall, from the significant findings, only higher levels in glutamatergic metabolite levels were reported (table 2), with Glu described in three studies (de la Fuente-Sandoval et al., 2013; de la Fuente-Sandoval et al., 2011; Plitman et al., 2016) and Glx in four studies (de la Fuente-Sandoval et al., 2013; de la Fuente-Sandoval et al., 2018; Goto et al., 2012; White et al., 2015).

In patients with FEP, higher levels of Glu were found in two dorsal caudate studies (de la Fuente-Sandoval et al., 2013; de la Fuente-Sandoval et al., 2011) and in one study of the associative striatum (Plitman et al., 2016), and higher levels of Glx were found in one study of the left basal ganglia (Goto et al., 2012) and in two of the dorsal caudate (de la Fuente-Sandoval et al., 2013; de la Fuente-Sandoval et al., 2018). In one study, higher levels of Glx were also found in the substantia nigra of patients with schizophrenia (White et al., 2015).

***Temporal Region***

Three of the five studies investigating the STG, STS, temporal cortex, and auditory cortex in patients with schizophrenia reported significant findings, all with medium effect sizes. For Glu, while one study reported on lower levels of the metabolite in the STG (Atagun et al., 2015) another reported on higher levels in the STS (Balz et al., 2018). One study investigating the temporal cortex found significantly lower levels of Glx in patients (Hugdahl et al., 2015).

***Hippocampus***

Fifteen studies reported on glutamatergic levels in FEP and in schizophrenia, of which significant differences in Glu and Glx levels were noted in five schizophrenia studies. While higher metabolite levels were noted in one study of Glu with a large effect size (Gallinat et al., 2016) and one Glx study with a medium effect size (Kraguljac et al., 2019), reduced levels were reported for Glu in two studies (Singh et al., 2018; Stan et al., 2015), with a small and large effect size, respectively, and for Glx in one study (Rauchmann et al., 2020) with a medium effect size.

***OCC***

Six studies assessed Glu and Gln levels in the OCC and in the visual cortex in patients with schizophrenia. Significant findings were reported in two studies of the OCC with medium effect sizes, with one study reporting on higher levels of both Glu and Glu (Wijtenburg et al., 2021b) and another reporting on lower levels on Glu (Thakkar et al., 2017).

***Thalamus***

From the ten studies investigating glutamatergic levels in the thalamus of patients with FEP and with schizophrenia, four schizophrenia studies reported significantly higher levels of Glu (Bojesen et al., 2021), with a small effect size, and Gln (Aoyama et al., 2011; Taylor et al., 2017; Théberge et al., 2003), with large effect sizes.

***Other Regions***

From the two studies that investigated the cerebellar cortex in patients with FEP, one reported a significant higher Glu levels (de la Fuente-Sandoval et al., 2013) with a medium effect size (d = 0.66). Higher levels of both Gln and Glx with medium effect were also found in one of three studies of the centrum semiovale of patients with schizophrenia (Wijtenburg et al., 2021a). One study investigating frontal WM, temporal WM, and occipital WM (Chang et al., 2007) reported on an overall elevated Glx levels in elderly patients with schizophrenia with a medium effect (d = 0.46).

Thirteen studies investigating the IFG, the insula, the parietal cortex, the POC, and frontal WM revealed no significant findings regarding changes in glutamatergic levels between participants and healthy controls. A comprehensive breakdown of reported directions of change can be found in Supplementary Table 3.

While Glx levels were found to be significantly higher in the medial frontal cortex of participants with schizophrenia in one study (Bartolomeo et al., 2019), another reported an significant decrease in the metabolite levels in the lateral prefrontal cortex (Ćurčić-Blake et al., 2017), both with medium effect size.

**Supplemental Figure 4**

*Statistical Significance for Glutamatergic Metabolites Between Brain Regions*

**
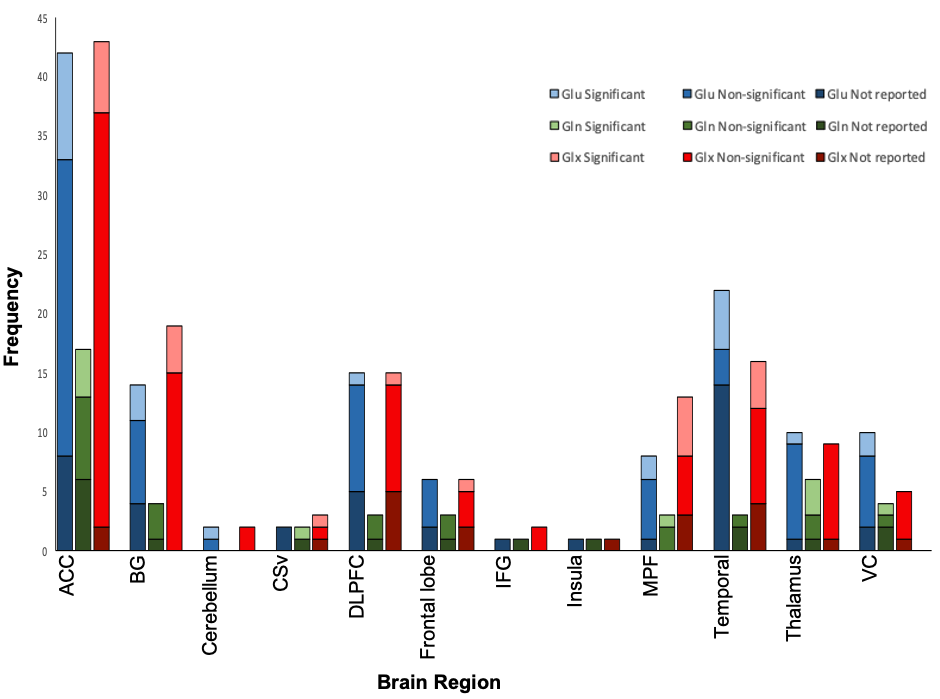
**

Statistical significance considered at α = 0.05.

Abbreviations: ACC, anterior cingulate cortex; BG, basal ganglia; CSv, centrum semiovale; DLPFC, dorsolateral prefrontal cortex; IFG, inferior frontal gyrus; Gln, glutamine; Glu, glutamate; Glx, glutamate+glutamine; MPF, medial prefrontal córtex; VC, visual cortex.
